# Strategies to improve outcomes of obstetric emergencies in sub-Saharan Africa: A scoping review

**DOI:** 10.1371/journal.pone.0355174

**Published:** 2026-09-01

**Authors:** Serah Wanjiru Wachira, Ruth Wagathu, Beth Waweru, Rose Maina, Abednego Ongeso, Constance Sibongile Shumba, Benard Daniel Mutwiri, Diana Mbattude, Cliff Asher Aliga, Lucy Kisaka, Peter Gatiti

**Affiliations:** 1 School of Nursing and Midwifery, East Africa, The Aga Khan University, Nairobi, Kenya; 2 Division of Epidemiology and Social Sciences, Institute for Health and Humanity, Medical College of Wisconsin, Milwaukee, Wisconsin, United States of America; 3 School of Nursing and Midwifery, East Africa, The Aga Khan University, Kampala, Uganda; 4 School of Nursing and Midwifery, East Africa, The Aga Khan University, Dar es Salaam, Tanzania; 5 Faculty of Health Sciences, Library, The Aga Khan University, Nairobi, Kenya; LMU München: Ludwig-Maximilians-Universitat Munchen, NEPAL

## Abstract

**Background:**

Obstetric emergencies remain a leading cause of maternal morbidity and mortality in sub-Saharan Africa (SSA) despite increased access to facility-based deliveries and emergency obstetric care. Improving outcomes requires not only life-saving clinical interventions but also effective health system strategies. This review mapped published evidence on strategies used to improve outcomes of obstetric emergencies in SSA.

**Objective:**

This scoping review aimed to identify strategies reported in the literature to improve outcomes of obstetric emergencies in SSA.

**Methods:**

The review was reported in accordance with PRISMA-ScR. A comprehensive electronic search of PubMed, CINAHL, SCOPUS, and CORE identified studies published between January 2019 and December 2025. Eligible studies focused on strategies to improve outcomes for pregnant women experiencing obstetric emergencies in sub-Saharan Africa and were published in English. Data were extracted using a standardized data-extraction tool and descriptively mapped through thematic analysis to identify the range and characteristics of strategies reported across studies.

**Results:**

Of the 728 records identified, 64 studies met the inclusion criteria. The identified strategies were organised into four interrelated themes. Three themes described strategies to improve outcomes of obstetric emergencies: (1) life-saving clinical and health system interventions, including pharmacological therapies, monitoring and triage tools, and service-strengthening initiatives; (2) health workforce education, training, and mentorship, which improved provider competence and selected process outcomes but showed variable effects on maternal mortality; and (3) health system organisation, referral mechanisms, and policy implementation, including emergency obstetric care protocols, referral coordination, and service decentralisation. A fourth theme described contextual factors influencing the implementation and effectiveness of these strategies, including infrastructure limitations, resource constraints, workforce shortages, and delays in accessing care.

**Conclusion:**

Improving outcomes of obstetric emergencies in sub-Saharan Africa requires integrated, context-responsive strategies that combine evidence-based clinical interventions with strengthened health systems. This review demonstrates that effective obstetric emergency care depends not only on life-saving clinical management but also on coordinated referral systems, a competent and supported health workforce, functional health system organization, and enabling implementation contexts. Future research should evaluate the effectiveness, scalability, and sustainability of integrated strategies across diverse resource-constrained settings.

## Introduction

Obstetric emergencies remain a leading cause of preventable maternal morbidity and mortality globally, with a disproportionate burden borne by countries in sub-Saharan Africa (SSA). SSA accounts for around 70% of global maternal deaths, despite progress in reducing maternal mortality since 2000 [1,2]. This high maternal mortality is driven by delays in the recognition of complications, timely referral, and receipt of appropriate care, factors widely conceptualised within the ‘three delays’ framework and consistently demonstrated in facility-based referral studies [3]. Maternal mortality in SSA is caused by obstetric emergencies, with obstetric haemorrhage, hypertensive disorders of pregnancy, maternal sepsis, obstructed labour, and complications of unsafe abortion accounting for a substantial proportion of maternal deaths [4]. These obstetric emergencies require timely and coordinated responses that extend beyond isolated clinical interventions to include skilled health workers, reliable commodities and blood supply, functional referral pathways, and responsive health system organisation [5–7]. Consequently, strategies to improve outcomes of obstetric emergencies should be understood as multidimensional, encompassing life-saving clinical interventions, workforce capacity-building, effective referral and transport mechanisms, and broader health-system and socio-structural determinants that shape access to timely, quality care.

Maternal mortality remains a major public health challenge, particularly in low- and middle-income countries (LMICs) [8–10], where improvements in obstetric care have not translated into significant reductions in maternal deaths. Despite sustained global efforts, the burden of maternal mortality remains disproportionately high in these settings. The Sustainable Development Goal (SDG) 3, specifically target 3.1, aims to reduce the global maternal mortality ratio to less than 70 per 100,000 live births by 2030 [11]. However, achieving this target requires a comprehensive understanding of the factors contributing to maternal mortality and the implementation of effective, context-appropriate interventions.

Effective management of the contributing factors depends on timely recognition and access to quality Emergency Obstetric and Newborn Care (EmONC). However, this remains a challenge in SSA due to shortages of critical health system resources, including emergency and critical care services. In 2017, the World Health Organization (WHO) estimated that approximately 295,000 women died globally due to pregnancy and childbirth-related causes, with nearly two-thirds of these deaths occurring in SSA [12]. More recent estimates indicate that in 2023, Despite being largely preventable, maternal deaths remain disproportionately concentrated in low- and middle-income countries (LMICs), where the maternal mortality ratio is approximately 346 per 100,000 live births compared with 10 per 100,000 live births in high-income countries [11,13]. The lifetime risk of maternal death remains starkly unequal, rising from approximately 1 in 7,933 in high-income countries to nearly 1 in 66 in low-income settings [12]. These disparities highlight the urgent need to strengthen obstetric emergency care in SSA.

Despite increased coverage of antenatal care, skilled birth attendance, and facility-based deliveries, maternal mortality in SSA remains unacceptably high. This apparent paradox highlights persistent gaps in the management of obstetric emergencies, particularly at the health system level. One major gap is the incomplete functionality of EmONC services [14]. Although many facilities are designated as Basic or Comprehensive EmONC (BEmONC/CEmONC) centers, they are often unable to consistently provide the full range of internationally defined signal functions, including administration of uterotonics and anticonvulsants, assisted vaginal delivery, neonatal resuscitation, blood transfusion, and caesarean section. This mismatch between facility designation and actual service readiness results in ‘name-only’ EmONC capacity, weakening referral systems and delaying life-saving interventions.

Health workforce constraints further undermine effective responses to obstetric emergencies. Persistent shortages of skilled midwives, obstetricians, anesthetists, and critical care providers, coupled with high staff turnover, task shifting without adequate supervision, and limited opportunities for continuing professional development, contribute to gaps in clinical competence at the point of care [15,16]. These challenges are strongly associated with delayed recognition and suboptimal management of obstetric emergencies. Additional gaps exist in clinical monitoring and adherence to established care protocols, particularly during labor and the immediate postpartum period. Inconsistent use of standardized labor monitoring tools, inadequate postpartum surveillance, especially within the first 24 hours after birth, and failure to recognize early warning signs contribute to delayed escalation of care and preventable maternal and neonatal deaths.

In response to these challenges, a range of strategies has been proposed and implemented to strengthen obstetric emergency care across different settings. These include expanding access to fully functional EmONC services and strengthening workforce capacity through Continuous Professional Development (CPD). Traditional midwives continue to play a crucial role in maternal and newborn care, particularly in rural and indigenous communities where access to formal health services is limited. Evidence suggests that culturally adapted tools, such as clinical flowcharts co-developed with midwives, can facilitate early recognition of obstetric complications and timely referral [17]. Additionally, CPD programmes, including simulation-based training, have been shown to enhance healthcare providers’ knowledge, skills, confidence, teamwork, and preparedness for managing high-risk obstetric emergencies [18]. Interdisciplinary collaboration has also been identified as a key strategy for improving coordination and continuity of emergency obstetric care [19].

Despite the growing availability of clinical and health-system strategies, evidence on their implementation, adaptation, effectiveness, and sustainability across SSA remains fragmented. Most published studies evaluate isolated interventions within specific facilities or countries, with limited examination of how multiple strategies interact across different levels of the health system or are adapted to diverse resource-constrained contexts. Consequently, there is limited understanding of which combinations of strategies are most effective, under what conditions they work, and how to implement and sustain them at scale to improve outcome of obstetric emergencies.

Although a growing body of literature has examined maternal mortality, emergency obstetric care availability, and individual interventions in SSA, existing reviews tend to focus on single dimensions of care, such as clinical management protocols, workforce training initiatives, or health system readiness in isolation. Few reviews have synthesised evidence across the full continuum of strategies required to improve outcomes in obstetric emergencies, particularly those that integrate life-saving clinical interventions with health workforce capacity, health system organisation, referral mechanisms, and contextual and systemic influences on care delivery. Unlike previous reviews that have focused on individual interventions or specific components of emergency obstetric care, this review adopts a comprehensive health-systems perspective by mapping the full range of strategies reported across the continuum of obstetric emergency management.

Furthermore, much of the existing literature predates recent policy and programmatic shifts and does not explicitly focus on obstetric emergencies as distinct, high-acuity events requiring coordinated responses across levels of care. To address this gap, this scoping review aimed to map and describe the available evidence on strategies used to improve outcomes of obstetric emergencies among pregnant and postpartum women in SSA. Specifically, the review sought to: [1] identify the range of clinical, workforce, referral, health system, and community-based strategies reported in the literature; [2] describe the contexts in which these strategies have been implemented; and [3] identify evidence gaps to inform policy, practice, and future research.

### Review question

What strategies have been reported in the literature to improve outcomes of obstetric emergencies in SSA?

## Methods

This scoping review is reported in line with the Preferred Reporting Items for Systematic Reviews and Meta-Analyses extension for Scoping Reviews (PRISMA-ScR) [20]. A scoping review approach was selected to map the breadth and nature of available evidence on clinical and health system strategies to improve outcomes in obstetric emergencies, and to identify key concepts, evidence gaps, and implementation contexts across diverse settings.

### Eligibility criteria

Eligibility criteria were defined using the Population–Concept–Context (PCC) framework.

### Inclusion criteria

***Population***: Studies involving pregnant or postpartum women experiencing obstetric emergencies, including but not limited to postpartum haemorrhage, hypertensive disorders of pregnancy, sepsis, obstructed labour, and other life-threatening obstetric complications.

***Concept***: Studies reporting clinical strategies (including but not limited to pharmacological interventions, supportive or critical care management, and use of monitoring tools) and/or health system strategies (including but not limited to workforce training and mentorship, referral and transport systems, service organisation, clinical protocols, or policy-related interventions) aimed at improving maternal outcomes during obstetric emergencies.

***Context***: Studies conducted in countries classified by the World Health Organization as SSA and published in English between January 2019 and December 2025.

***Study design***: Empirical studies and relevant evidence-informed conceptual or framework papers describing, evaluating, or informing strategies to improve outcomes of obstetric emergencies in SSA were eligible for inclusion.

### Exclusion criteria

Studies were excluded if they:

i Did not focus on obstetric emergencies
ii Were purely descriptive or epidemiological without reporting an intervention or strategy
iii Were conducted outside SSA
iv Were published in languages other than English
v Were conference abstracts, commentaries, editorials, or review articles

### Search strategy

**Fig 1:**
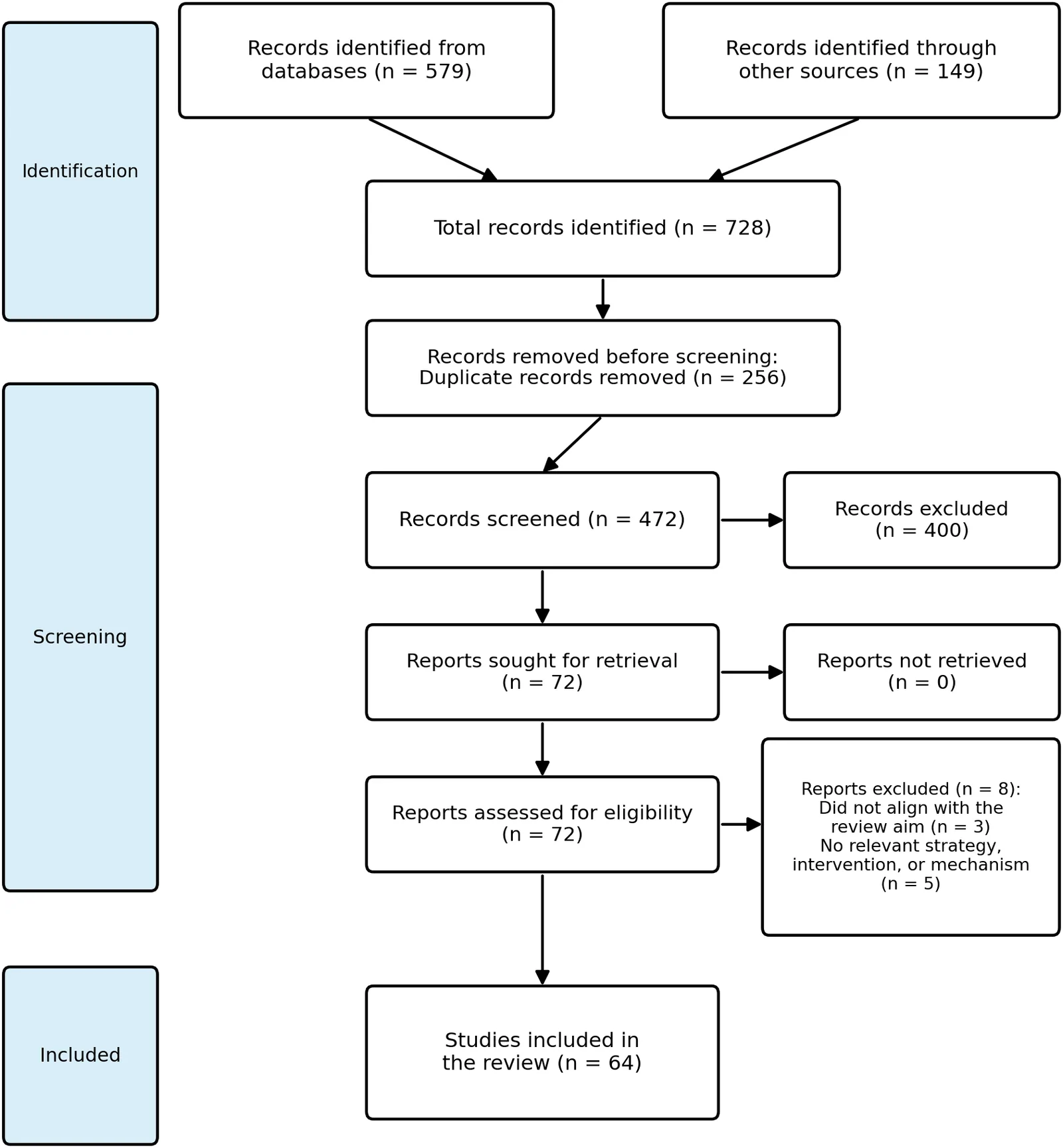
PRISMA flow diagram of the study-selection process.

A comprehensive electronic search was conducted in PubMed, CINAHL, Scopus, and CORE to identify studies published from January 2019 to December 2025. Database-specific search strategies combined controlled vocabulary (e.g., MeSH terms) with free-text keywords, using Boolean operators (AND/OR). Core search concepts included obstetric emergencies (e.g., postpartum hemorrhage, hypertensive disorders of pregnancy, obstetric sepsis, obstructed labor), strategies or interventions (e.g., emergency obstetric care, referral systems, workforce training, clinical protocols, critical care), and SSA. The complete search strategies for each database are provided in S1 File.to facilitate reproducibility. In addition to database searches, reference lists of included studies were manually screened to identify further relevant publications. The search included empirical studies and relevant evidence-informed conceptual or framework papers, with no restrictions on study design. The study selection process followed the Preferred Reporting Items for Systematic Reviews and Meta-Analyses extension for Scoping Reviews (PRISMA-ScR) and is illustrated in the PRISMA flow diagram (Fig 1).

### Study selection

Following the database search, titles and abstracts of retrieved studies were downloaded and exported into EndNote for reference management. Deduplication was conducted by PG. Before commencing study selection, all reviewers participated in an online calibration meeting to review the screening tool, discuss the Population–Concept–Context (PCC)-based eligibility criteria, and ensure a shared understanding of the inclusion and exclusion criteria prior to screening. The review team was then organised into four pairs of independent reviewers ([SW, BW], [RW, CA], [RM, DM], and [LK, AO]) to screen titles and abstracts against the predefined inclusion and exclusion criteria. Studies deemed potentially eligible were advanced to full-text screening. Any discrepancies arising during either stage of screening were resolved through discussion and consensus between reviewers. Where consensus could not be reached, eligibility decisions were resolved through consultation with a third independent reviewer (BM). Only studies meeting the inclusion and exclusion criteria were included in the final review.

### Data extraction and quality assessment

The review team utilized the PCC Framework to validate the data extraction design and approach [20,21]. A standardised data extraction tool was used, and characteristics of interest were extracted. Two [2] independent reviewers ([SW, BW], [RW, CA], [RM, DM], [LK, AO]) conducted data extraction from the eligible studies included in the final review. The reviewers jointly harmonized the extracted data through mutual consensus, with the third reviewer (BM) providing their opinion to inform the final decision. The extracted data included the study objective or aim, author(s), year of publication, country, study design, study population or sample, intervention or strategy, implementation context, and reported maternal or obstetric outcomes.

### Quality assessment

Consistent with established scoping review methodology, a formal methodological quality assessment (critical appraisal) of the included studies was not undertaken. The primary purpose of this review was to map the breadth and characteristics of the available evidence rather than to evaluate the methodological quality of individual studies. In line with the Joanna Briggs Institute guidance and the PRISMA-ScR reporting framework, no studies were excluded for methodological quality. During study selection, eight reviewers (SW, BW, RW, CA, RM, DM, LK, and AO) independently screened titles, abstracts, and full-text articles against the predefined eligibility criteria to minimise selection bias. Any disagreements regarding study eligibility were resolved through discussion until consensus was reached.

### Strategy for mapping the evidence

Extracted data were descriptively mapped to identify the range, characteristics, and implementation contexts of strategies reported across the included studies. An inductive thematic analysis was undertaken to organize the mapped evidence into conceptually related categories representing the major types of strategies identified [22]. The mapping process involved iterative review and refinement of the extracted data by two reviewers (PG and SW), who collaboratively grouped similar strategies into overarching themes through discussion and consensus. The mapped evidence is presented using descriptive tables, figures, and narrative summaries that address the review question and objectives.

## Results

The comprehensive search identified 728 records from PubMed, CINAHL, Scopus, CORE, and manual reference list screening. After removal of 256 duplicate records, 472 unique records underwent title and abstract screening. Of these, 400 records were excluded for not meeting the eligibility criteria, and 72 full-text articles were assessed for eligibility. Eight full-text articles were excluded because they did not align with the review objective or did not report a strategy, intervention, or mechanism aimed at improving outcomes of obstetric emergencies. Consequently, 64 studies met the eligibility criteria and were included in this scoping review (Fig 1).

### Characteristics of the included studies

A total of 64 studies published between 2019 and 2025 met the eligibility criteria and were included in this scoping review. The studies represented a broad range of methodological designs, including qualitative, cross-sectional, cohort, randomized controlled, mixed-methods, implementation, observational, and retrospective studies. The studies were conducted across multiple countries in SSA and encompassed diverse healthcare settings, including primary healthcare facilities, district hospitals, referral hospitals, tertiary hospitals, and community settings.

The included studies examined strategies targeting a range of obstetric emergencies, most commonly postpartum hemorrhage, hypertensive disorders of pregnancy, maternal sepsis, obstructed labor, and pregnancy-related critical illness. Reported strategies included life-saving clinical interventions, health workforce education and mentorship, referral and transport systems, health system strengthening initiatives, implementation of clinical protocols, quality improvement interventions, and policy or governance approaches. Most studies reported process outcomes, such as provider competence, referral performance, timeliness of care, implementation outcomes, and service readiness, whereas comparatively fewer studies evaluated maternal mortality, severe maternal outcomes, or maternal near-miss as primary endpoints. The characteristics of the included studies are presented in Table 1.

**Table 1:** Characteristics of the included studies.

#	Study	Aim(s)	Country	Study Design	Population	Intervention	Outcome(s)
1.	Daniels and Abuosi 2020 [23]	To identify barriers associated with the referral of emergency obstetric cases to the leading national referral centre.	Ghana	Qualitative method	31 patients, 34 health care providers, and 24 patients’ relatives	Referral of patients from lower levels of care to higher levels.	Barriers included communication, referral, transportation system, health infrastructure, supplies, and human resource constraints.
2.	Banke-Thomas, Wong 2021 [24]	To derive and compare estimates of travel time to reach comprehensive emergency obstetric care (CEmOC).	Nigeria	Retrospective study	732 pregnant women	Comparison of estimates of travel time to reach CEmONC.	Large-scale national and global models may not be effective for advocacy and service planning. Web-based platforms capable of capturing relevant, context-specific data on travel and real-time traffic are critical for effective service planning.
3.	Moore, Thomson,2019 [25]	To introduce a modified obstetric early warning system and assess its feasibility and potential impact on improving postoperative monitoring and earlier identification and management of deteriorating patients.	Ethiopia	Pilot study (Interventional)	20 nurses	Introduction of a modified obstetric early warning system, staff training.	Early identification and management of deteriorating post-op obstetric patients.
4.	Nabulo, Gottfredsdottir, 2023 [26]	To explore health system and client related factors to understand how these could have facilitated or hindered the referral process.	Uganda	An exploratory qualitative study.	Postnatal women and attendants as key informants.	Training HCPs in respectful maternity care (RMC) may improve quality of care and foster positive postnatal client experiences. Refresher sessions on obstetric referral procedures for HCPs are suggested.	The experience of obstetric referral for women was largely unpleasant due to delays and poor quality of care which contributed to perinatal mortality and maternal morbidities.
5.	Bekele, Fikre, 2022 [27]	To assess the factors affecting the utilization of anti-shock garments among obstetric care providers in public hospitals.	Ethiopia	Facility-based cross-sectional study	403 obstetric care providers	A nonpneumatic antishock garment utilization.	Training about nonpneumatic antishock garment (NASG), availability of NASG, availability of protocol, respondents’ attitude, and knowledge improved utilization leading to reduction in maternal mortality.
6.	Dominico, Serbanescu, 2022 [28]	To evaluate the outcome and impact of the comprehensive approach to improving Emergency Obstetric and Newborn Care program.	Tanzania	Implementation study	127 health facilities	Decentralize Comprehensive EmONC from hospitals to health centers.	The decentralization of comprehensive EmONC, mainly provided by associate clinicians and nurses, resulted in noticeable enhancements in the accessibility and use of life-saving birth care in Kigoma leading to reduction of MMR.
7.	Yeshitila, Bante, 2021 [29]	To assess utilization of non-pneumatic anti-shock garment to control complications of post-partum hemorrhage and associated factors among obstetric care providers.	Ethiopia	A facility-based cross-sectional study	412 obstetric health care providers	Application of NASG to women with obstetric hemorrhage.	Adequate knowledge, training, and a positive attitude improved utilization of non-pneumatic anti-shock garment.
8.	Sevene, Boene, 2021 [30]	To describe the feasibility of task-sharing, the initial screening and initiation of obstetric emergency care for pre-eclampsia/eclampsia.	Mozambique	Mixed methods (qualitative and quantitative)	56 health facilities	Task sharing, the initial screening and initiation of obstetric emergency care.	Task-sharing for screening and pre-referral management of pre-eclampsia and eclampsia was considered viable and well-received at the community level.
9.	Unwaha, Bello, 2020 [31]	To assess the effectiveness of a 12- hour versus 24- hour intravenous maintenance dose of magnesium sulfate in women with pre- eclampsia, and the maternal and fetal outcomes.	Nigeria	A randomized controlled trial	80 pregnant women	Use of magnesium sulfate in management of severe pre-eclampsia.	A 12-hour maintenance dose of intravenous MgSO4 for managing severe pre-eclampsia is as effective and safe as the traditional 24-hour maintenance dose.
10.	Marotta, Di Gennaro, 2020 [32]	To explore the value-based dimension by performing a cost-utility analysis concerning the implementation and one-year operation of the HDU.	Siera Leone	Retrospective cost-utility review	All women during pregnancy or up to 42 days after the termination of pregnancy admitted to the hospital, and HDU	Healthcare infrastructure implementation and costing for obstetric critical care.	General costs of care and cost of care per patient, cost-utility analysis, value assessment, and primary endpoints (quality-adjusted life-years) of patients admitted to the HDU during the study period.
11.	Kinnevey, Douglas, 2021 [33]	To propose a method for teaching the full HBS curriculum.	Uganda	Quasi-experimental pre- and post-study design	Skilled birth attendants (midwives) from 19 rural Ugandan prenatal clinics	Training program evaluating the effectiveness of a model for teaching HBS courses among rural midwives.	Learners’ self-assessment of confidence and knowledge in the diagnosis and management of common maternal and neonatal complications and self-efficacy in the diagnosis and management of peripartum conditions.
12.	King, Tarway-Twalla, 2022 [34]	To assess the quality of childbirth care in Liberia, focusing on readiness for emergency care and referral, staffing, and the volume of births in health facilities.	Liberia	Secondary data analysis of the DHS surveys	All health facilities in Liberia	Percentage increase in the number of live births between 2004 and 2017 with the biggest rise in public clinics in rural health facilities.- The proportion of facilities able to provide BEmONC signal functions.- Percentage of facilities with a skilled birth attendant.	Despite the increase in facility births, only 18% of facilities could perform basic emergency obstetric and neonatal care (EmONC), and just 8% could provide blood transfusions and cesarean sections. Moreover, 63% of facility births occurred in places without full basic emergency readiness, and 60% of facilities were unable to make emergency referrals.
13.	Kaselitz, James, 2019 [35]	To explore basic and comprehensive emergency obstetric service provision across four districts in rural northern Ghana, and whether women were more likely to deliver at facilities with more skilled care.	Ghana	Prospective	95 health facilities were identified across the four study districts in Ghana	Description of Health care infrastructure for Basic Emergency Neonatal and Obstetric Care including staffing of health units, availability of services, and how many women presented with complications in those facilities.	Availability and performance of facilities for BEMONC and CEMONC at different levels of health facilities.
14.	Nathan, Seed, 2019 [36]	To evaluate shock index (SI) thresholds as predictors of outcomes in obstetric patients.	South Africa	prospective cohort study	283 Women diagnosed with PPH.126 Women diagnosed with maternal sepsis.	The “first” and “worst” SI following diagnosis were recorded, compared with conventional vital signs, and evaluated.	In hemorrhage, risk of all outcomes increased with increasing “first” SI. In sepsis, risk of all outcomes increased with increasing “worst” SI.
15.	Nyamtema, LeBlanc, 2022 [37]	To study how to improve access to CEmONC services in underserved rural areas.	Tanzania	A five-year longitudinal cohort study	Seven health centres and 21 satellite dispensaries in Morogoro region.	Capacity building in emergency obstetric and newborn care and anesthesia.	Mean monthly deliveries increased from 183 to 358 during the intervention period. The referral rate to district hospitals decreased from 6.0% to 4.0% during the intervention period. The obstetric case fatality rate decreased slightly from 1.5% to 1.1%.
16.	Mpunga Mukendi, Chenge, 2019 [38]	To assess the availability, quality and equity of emergency obstetric care (EmOC) in the DRC.	Democratic Republic of the Congo	A cross-sectional survey	Health facilities	Resulted in unresponsive EMCOR services with mothers receiving inadequate interventions sometimes after major delays across different levels of care. Future reforms should align policies to implementation contexts and resources for optimal results.	The distribution and quality of EmOC was problematic.
17.	Nyamtema, Scott, 2022 [39]	To determine the causes and assess the factors that contributed to the maternal deaths based on the “three delays model”.	Tanzania	Mixed-methods approach.	42 clinicians from five health centres	26 associate clinicians from 5 health centres were trained in CEmONC and anaesthesia.	The met need for emergency obstetric care increased significantly from 45% at baseline to 119% during the intervention period. The met need for emergency obstetric care in the control group also increased from 53% to 77%.
18.	Okonofua, Ntoimo, 2019 [40]	To assess the existing knowledge and skills relating to Emergency Obstetrics Care among health providers in eight referral maternity hospitals in Nigeria.	Nigeria	Cross sectional	341 health providers (148 doctors and 193 nurses/ midwives)	Participants knowledge and skills in offering specific EMOC services, and their confidence in transferring the skills to mid-level providers.	Health providers scored less than 46% in a composite EMOC knowledge score. Doctors scored considerably higher than the nurses/midwives.
19.	Ramavhoya, Maputle, 2020 [41]	To explore and describe the experiences of midwives in managing women diagnosed with hypertensive disorders during pregnancy in rural areas.	South Africa	Qualitative, descriptive phenomenological	18 midwives from primary health care facilities in the Mopani and Vhembe districts	Invasive mechanical ventilation, blood transfusion, inotropics/vasopressors support, re-operation, hemodialysis	High Maternal Mortality Rate and influencing factors like; poor quality care, inadequate transport, and lack of skills.Training and Guidelines and Medical Protocols.Decline in Maternal Deaths and Challenges in Adherence.
20.	Rudakemwa, Cassidy, 2021 [42]	To assess reasons for ICU admission and accuracy of prediction models for mortality of obstetric patients	Rwanda	Prospective	94 obstetric patients in ICU	3-month specialized training program for healthcare providers in five rural healthcare facilities.	Sepsis (31.9%) and obstetric hemorrhage (25.5%) were the most common reasons for ICU admission.Identified gaps: there is a need for larger sample sizes, long-term follow-up, improved ICU capacity, validation of predictive tools, and adaptations for resource-limited settings.
21.	Mselle, Sirili, 2021 [43]	To explore how healthcare professionals, managers and community members experienced the implementation of a training program in comprehensive emergency obstetric and neonatal care training in rural Tanzania	Tanzania	Qualitative	24 focus group discussions	Utilization of MEOWS and qSOFA scores	Increased skills and confidence in healthcare teams, improved community trust, and a reduction in maternal and neonatal.
22.	Pisani, De Nicolo, 2020 [44]	To determine the frequency, timing, and type of pulmonary complications in critically ill parturient	Sierra Leone	Prospective observational study	166 critically ill obstetric patients	Lung ultrasound (LUS) on admission.	21% had pulmonary complications. Complications were associated with poor outcomes.
23.	Prin, Kadyaudzu, 2019 [45]	To characterize ICU utilization for obstetric patients and its relationship with in-hospital mortality.	Malawi	Prospective observational cohort study	105 obstetric patients	ICU utilization for obstetric patients.	23% of ICU admissions were obstetric. High in-hospital mortality rate of 49%. The majority required mechanical ventilation (95%) and vasopressors (48%).
24.	Rosenberg, Williams, 2021 [46]	To improve maternal mortality rates by implementing a prehospital Emergency Obstetrics and Neonatal Course.	Rwanda	Cohort Study	20 participants	EONC training and train-the-trainers program.	Significant improvement in scores: EONC1 median scores increased from 60% to 92%, and EONC2 from 52% to 96%.
25.	Pattinson, Bergh, 2019 [47]	To determine whether there was a change in the number of maternal deaths and in the iMMR over time that could be attributed to the training of healthcare professionals.	South Africa	Before-and-after study	3237 healthcare professionals were trained at 346 workshops	Off-site skills-and-drills training in EmOC.	Reductions of 29.3% in the number of maternal deaths and 17.5% in the number of maternal deaths from direct obstetric causes.
26.	Mukuru, Kiwanukay, 2021 [48]	To explore the barriers frontline implementers of EmOC policies faced, their coping behaviours and the consequences for maternal health.	Uganda	A retrospective exploratory qualitative study	8 in-depth interviews with doctors and 17 midwives in health facilities	Frontline implementers’ coping behaviours oftentimes involved improvization leading to delivery of incomplete and inconsistent EmOC service packages.	Unresponsive EmOC services with mothers receiving inadequate interventions sometimes after major delays across different levels of care.
27.	Mwaniki, Edwards, 2020 [49]	To evaluate the completeness of the maternal death review (MDR) process, focusing on aspects such as notification, audit completion, documentation quality, causes of maternal deaths, hospital-related delays, missed opportunities, and action points in seven hospitals within Thika sub-county, Kenya.	Kenya	A retrospective design	A review of 43 maternal deaths	Enhanced monitoring and evaluation of maternal deaths including revision of MDR forms to capture informative data.	Community based maternal death reporting and reviews are not routinely done thus contributes to the overall underreporting.
28.	Akter, Forbes, 2022 [50]	To explore healthcare providers’ knowledge and practices of PPH detection and management after vaginal birth.	Kenya, Nigeria, and South Africa	Qualitative	45 maternity healthcare providers of nine hospitals in Kenya, Nigeria, and South Africa.	Enhancing in-service training and implementing objective-evidence based blood loss measurement tools to facilitate better PPH management.	Improving in-service training and adopting objective methods for measuring blood loss.
29.	Anto-Ocrah, Cushman, 2020 [51]	To propose an access framework that integrates the Three Delays Model in maternal health with emergency care (EC) interventions to improve timely access to emergency obstetric care and reduce maternal mortality in SSA.	SSA	Conceptual analysis	N/A	Policy framework to address each of the three delays in emergency obstetric care.	Proposed access framework, which aims to bridge maternal health service delivery with emergency medicine.
30.	Aikpitanyi, Ohenhen, 2019 [52]	To identify the medical causes and contributory factors of maternal mortality through the MPDSR process and to elucidate the policy responses following the dissemination of the results.	Nigeria	Observational retrospective cohort study.	18 maternal deaths review.	Establishment of Maternal and Perinatal Death Surveillance and Response Committee.	Improved quality of care and a significant decline in the maternal mortality ratio at the referral hospital.
31.	Alobo, Ochola, 2021 [53]	To explore and describe the pathways for women requiring emergency obstetrics and newborn care and understand delays in accessing care after reaching a health facility.	Uganda	Qualitative study using critical incident technique and key informant interviews.	49 purposively selected health workers, patients, and attendants in northern Uganda.	Mapping pathways for maternal deaths and near-misses, interviews with health workers, patients, and attendants.	Pathways to emergency obstetric and newborn care, causes of delays in care, and the impact on maternal and neonatal outcomes.
32.	Heitkamp, Seinstra, 2019 [54]	To determine incidence, risk indicators, and outcomes of emergency peripartum hysterectomy in Metro East, Cape Town, South Africa.	South Africa	A prospective descriptive study design	59 women with Emergency peripartum hysterectomy	Hysterectomy arising from Emergency peripartum hemorrhage or sepsis.	The incidence of EPH for sepsis was higher than previously reported. Repeat cesarean was strongly associated with EPH. Clinical characteristics of sepsis-related EPH compared unfavorably with those of hemorrhage-related EPH.
33.	Kabo, Orobaton, 2019 [55]	To evaluate the impact of quality improvement interventions for obstetric emergencies following a baseline assessment.	Nigeria	A prospective intervention study	21 hospitals designated as comprehensive EmOC centers and 38 primary healthcare centers- Basic EmOC centres.	Quality improvement interventions based on baseline assessment.	The rise in facility-based births and met need for EmOC suggests increased availability, access, and utilization of EmOC.
34.	Jejaw, Debie, 2021 [56]	To evaluate the implementation of comprehensive emergency obstetric and newborn care program in terms of availability, compliance and acceptability dimensions at University of Gondar Comprehensive Specialized Hospital, Northwest Ethiopia.	Ethiopia	A single case study mixed method design	265 mothers who gave birth at University of Gondar, 13 key informant interviews, 49 non-participatory observations, 320 retrospective document reviews.	Comprehensive emergency obstetric and newborn care program.	The implementation status of the CEmONC program was good. Compliance with guidelines needed improvement. Essential emergency drugs and equipment were often stocked out.
35.	Germossa, Wondie, 2022 [57]	To investigate the availability of CEmONC signal functions and describe lessons learned from Transform Health support in Developing Regional State in Ethiopia.	Ethiopia	Cross-sectional study	15 public hospitals in four developing regions of Ethiopia.	Clinical mentorship	Availability of CEmONC signal functions improved, but maternal and neonatal mortality remained unchanged, indicating a need for further investigation and quality assessments.
36.	Dougherty, Gobezayehu, 2023 [58]	To determine Amhara’s clinical readiness and quantify the relationship between SF (signal function) and CC (clinical cascade) estimates of readiness to manage obstetric emergencies	Ethiopia	A prospective, cross-sectional, facility-based analysis	20 hospitals	Saving Little Lives Program Measuring facility readiness to manage basic obstetric emergencies to reduce MMR.	Discrepancy between SF and CC readiness classifications. Facilities unprepared to manage common obstetric emergencies. Potential difficulty in supply management for employees.
37.	Gonte, Peifer, 2022 [59]	To investigate the impact of the PPH Emergency Care (EmC) package in a low-resource setting.	Kenya	qualitative	14 individual interviews with clinical staff had implemented PPH EmC package, selected from 12 facilities where PPH EmC had previously been implemented	Implementation of the PPH EmC bundle package	Implementation of PPH EmC was feasible and positively impacted PPH emergency care, impacted facility and health system preparedness, referral coordination, teamwork, communication, and overall capacity to provide quality PPH emergency care with sustainability ensured by low cost and local partner support.
38.	Geleto, Chojenta, 2020 [60]	To assess the incidence of maternal near miss and contributing factors among hospitals in Ethiopia.	Ethiopia	Secondary analysis of National Dataset from the Ethiopian Public Health Institute	Maternal health indicators including obstetric complications, maternal deaths and births conducted at all hospitals available in Ethiopia.	–	Key maternal health indicators were: (i) total number of women admitted with direct and indirect causes of maternal mortality, (ii) proportion of women who died of direct causes, (iii) number of women who experienced near misses, and (iv) near miss incidence ratio and mortality index.
39.	Cavallaro, Benova, 2020 [61]	To establish where women deliver in Senegal and how this has changed over time. Readiness for treating obstetric complications and referring women with complications, according to facility level and region.	Senegal	Descriptive cross-sectional study (retrospective)	Chart review	Training interventions to include EmONC readiness through equipment availability and regular training and supervision at all health system levels, particularly health posts in the short term and health centres as deliveries shift to this facility level.	Investment in human resources is needed, particularly in rural facilities.
40.	Teka, Yemane, 2022 [62]	To examine the prevalence of maternal morbidities and deaths.	Ethiopia	Cross-sectional study	691 pregnant women or those who are within 42 days postpartum/any form of pregnancy termination.	• Prevention and treatment of severe PPH • Anticonvulsants for eclampsia • Prevention of cesarean section-related infection • Treatment for sepsis • Laparotomy for ruptured uterus • Corticosteroids for fetal lung maturation.	Primary outcome: Maternal Near Misses Secondary outcomes: Maternal Mortality Rate, Severe Maternal Outcome (SMO) and Mortality Index.
41.	Thwala, Blaauw, 2019 [63]	To identify health system enablers and barriers to the delivery EmOC from the perspective of district managers.	South Africa	A qualitative research design	19 district managers in charge	Coordination of EmOC services by managers.	Weak capacity of the health system to support the delivery of emergency obstetric care including limited staff, high leadership turnover, inadequate infrastructure, and funding.
42.	Mislu, Seid, 2023 [64]	To assess the magnitude of maternal third delay and associated factor among women admitted for emergency obstetric care.	Ethiopia	Descriptive cross sectional	542 women admitted for emergency obstetric care, systematic sampling method.	Access to emergency obstetric health care.	Delayed access to emergency obstetric care: 29.3% of the respondents experienced maternal third delay. There is need for health system strengthening to reduce delay.
43.	Ameyaw, Amoah, 2020 [65]	To assess the resources available for managing comprehensive emergency obstetric and neonatal care (CEmONC) and referral services.	Ghana	Cross-sectional survey	Maternity facilities in ten hospitals	Implementing financial and non-financial incentives to attract healthcare professionals to the region, mobilizing resources to improve essential equipment availability, and strengthening the health system to meet required standards.	The study concludes that the management of emergency obstetric care and referrals in Northern Ghana is severely hampered by limited human resources and equipment.
44.	Ayawine and Atinga, 2022 [66]	To explore user and community responses to service delivery gaps in emergency obstetric care provision in rural Ghana.	Ghana	Qualitative case study design grounded within a constructivist paradigm.	17 clients, 21 health providers, 6 focus groups (52 participants total)	Community interference in care processes, reliance on unskilled providers, local oxytocin use, non-compliance to treatment, mistrust in providers.	Coping mechanisms and behaviors of the community in response to gaps in service delivery and their impact on maternal health outcomes.
45.	Geleto, Chojenta, 2020 [67]	To investigate midwives’ perceptions about the quality of emergency obstetric care provided at hospitals in the Harari region of Ethiopia.	Ethiopia	Qualitative, Explanatory qualitative design.	12 midwives working in maternity units	Training opportunities for midwives to improve their skills.	Inadequate number of midwives, insufficient training opportunities for midwives to improve their skills and ineffective communication between patients and caregivers due to language barriers.
46.	Fagan et al., 2024 [68]	Assess ambulance service for emergency obstetric care.	Togo	Descriptive, retrospective	Pregnant/postpartum women (N = 2,926)	Free, 24/7 rural ambulance service integrated into primary care; training for drivers and health workers; community engagement to identify emergencies.	Access to emergency obstetric care rose from 18% in 2020 to 66.5% in 2022; improved linkages between communities and formal care.
47.	Beza et al., 2024 [69]	Review HDU admissions, diagnoses, interventions, and outcomes.	Ethiopia	Retrospective, observational	Obstetric HDU patients (N = 370)	Established a dedicated obstetric HDU; trained staff in critical and obstetric care to handle complex maternal cases.	Maternal mortality at 1.4%; only 0.42% required ICU transfer; most patients managed within HDU, reducing ICU burden.
48.	Adebayo et al., 2024 [70]	Determine PPH burden, risk factors, and outcomes.	Nigeria	Cross-sectional, secondary analysis	Women at 54 hospitals (N = 68,754)	Used uterotonics for PPH management; enhanced skilled care and hospital data systems for monitoring outcomes.	PPH affected 3.2% of women; 4.8% maternal deaths among PPH cases; stillbirth rate was 25%; gaps in care persisted.
49.	Gakwerere et al., 2024 [71]	To evaluate access to continuous professional development for capacity building among nurses and midwives providing emergency obstetric and neonatal care.	Rwanda	Cross-sectional, descriptive	Nurses and midwives (N = 149)	Continuous professional development (CPD) workshops; mentorship; policy on CPD credits.	79.9% failed to meet CPD requirements; significant gaps in EmONC knowledge noted; mentorship showed potential.
50.	Shikuku et al., 2024 [72]	Explore experiences with updated EmONC curriculum.	Kenya	Nested qualitative	Educators, mentors, students (N = 174)	App-based videos and simulation-based curriculum introduced with faculty mentorship and structured teaching.	Educator skills and student knowledge improved; resource and faculty shortages have a limited impact.
51.	Shannon et al., 2024 [73]	Assess danger sign awareness and barriers	Gambia	Cross-sectional survey	Women pregnant in last 5 years (N = 100)	Assessed danger sign knowledge; proposed mobile-based education to address gaps.	Actual knowledge low despite high self-perceived awareness; strong interest in digital health education.
52.	Birabwa et al., 2024 [74]	Analyze pathways to emergency obstetric care	Uganda	Cross-sectional	Women 15–49 yrs (N = 433)	Referral strengthening; EmONC access	Referral delays; stillbirths; multi-step access issues.
53.	Reynolds et al., 2024 [75]	Evaluate the mobile obstetric referral system (MORES)	Liberia	Qualitative	Health workers (N = 62)	Mobile Obstetric Referral System (MORES) using triage training and WhatsApp communication for emergency coordination.	Enhanced triage and communication; reduced care delays; improved readiness and accountability among providers.
54.	Zewde et al., 2024 [76]	Assess the impact of WHO NMCR on care and outcomes	Eritrea	Interrupted time series	Women with life-threatening conditions (N = 4,365)	Used WHO NMCR to review severe cases; applied QA cycles and trained staff for maternal care audits.	Significant reduction in critical maternal outcomes and delays; better decision-making through structured reviews.
55.	Abraha et al., 2025 [77]	To assess adverse birth outcomes and associated factors among mothers with antepartum hemorrhage	Ethiopia	Facility-based cross-sectional study	Mothers diagnosed with antepartum hemorrhage (309 records)	Emergency obstetric management of antepartum hemorrhage, including surgical intervention and referral care	Adverse birth outcomes, including preterm birth, low birth weight, stillbirth, and NICU admission
56.	Abdullahi et al., 2025 [78]	To compare maternal near-miss incidence and outcomes between secondary and tertiary healthcare facilities	Nigeria	Prospective observational study	Women with life-threatening obstetric complications (2,136 live births)	Emergency obstetric care provision at secondary and tertiary levels, including referral management	Maternal near-miss incidence, maternal mortality, mortality index, facility-level outcome differences
57.	Tarimo et al., 2025 [79]	To examine barriers to skilled birth attendance and emergency obstetric care using a Right to Health framework	Tanzania	Qualitative study	Pregnant and postpartum women in rural communities	Access to emergency obstetric care within existing health system structures	Barriers related to availability, accessibility, acceptability, and quality of emergency obstetric services
58.	Bulamba et al., 2025 [80]	To determine the incidence, risk factors, and outcomes of postpartum hemorrhage at a regional referral hospital	Uganda	Prospective observational cohort study	Women delivering at a regional referral hospital (2,358 deliveries)	Management of postpartum hemorrhage and facility readiness for emergency obstetric care	Postpartum hemorrhage incidence, maternal mortality, gaps in treatment availability and escalation
59.	Govender et al., 2025 [81]	To assess emergency care providers’ knowledge, attitudes, and practices related to obstetric hemorrhage management	South Africa	Cross-sectional survey	Emergency care providers (417 participants)	Provider knowledge, attitudes, and practices regarding obstetric hemorrhage management	Levels of knowledge, attitudes, and reported practices related to emergency obstetric hemorrhage care
60.	Sengoka et al., 2025 [82]	To explore the lived experiences of women who survived severe maternal complications	Tanzania	Descriptive qualitative study	Maternal near-miss survivors (12 women)	Care experiences related to management of severe maternal complications	Physical, psychological, social, and economic consequences of severe maternal morbidity
61.	Lunda et al., 2025 [83]	To determine maternal mortality ratios and avoidable factors before and during the COVID-19 period	South Africa	Retrospective cross-sectional study	Maternal death records from a tertiary hospital (57 cases)	Emergency obstetric and critical care service provision within a tertiary facility	Institutional maternal mortality ratio, causes of death, and avoidable patient, provider, and system factors
62.	Keem et al., 2025 [84]	To explore women’s experiences of referral following obstetric emergencies	Uganda	Qualitative phenomenological study	Women referred for emergency obstetric care (17 participants)	Referral processes and inter-facility care pathways during obstetric emergencies	Barriers and facilitators to referral, communication quality, and perceived quality of care
63.	Cooper et al., 2025 [85]	To assess whether antenatal risk stratification predicts adverse obstetric outcomes at delivery	Kenya	Prospective observational cohort study	Pregnant women receiving antenatal care (19,653 participants)	Antenatal risk stratification and delivery care processes	Adverse obstetric outcomes, delivery complications, predictive performance of risk stratification
64.	Teka et al., 2025 [86]	To determine the prevalence, clinical profile, and outcomes of obstructed labor	Ethiopia	Retrospective cross-sectional study	Women diagnosed with obstructed labor (83 cases)	Management of obstructed labor and referral pathways	Maternal complications, perinatal outcomes, referral patterns, and delays in care

The included studies were published between 2019 and 2025 and demonstrated a relatively consistent distribution across the review period. Although the number of publications varied slightly by year, evidence on strategies to improve outcomes of obstetric emergencies was available throughout the study period, indicating continued research activity in this area. The distribution of studies by year of publication is presented in Table 2.

**Table 2:** Publications selected for the study.

Year published	No.	%
2019	11	17.2
2020	11	17.2
2021	10	15.6
2022	9	14.1
2023	4	6.3
2024	9	14.1
2025	10	15.6
**Total**	**64**	**100.0**

**Fig 2:**
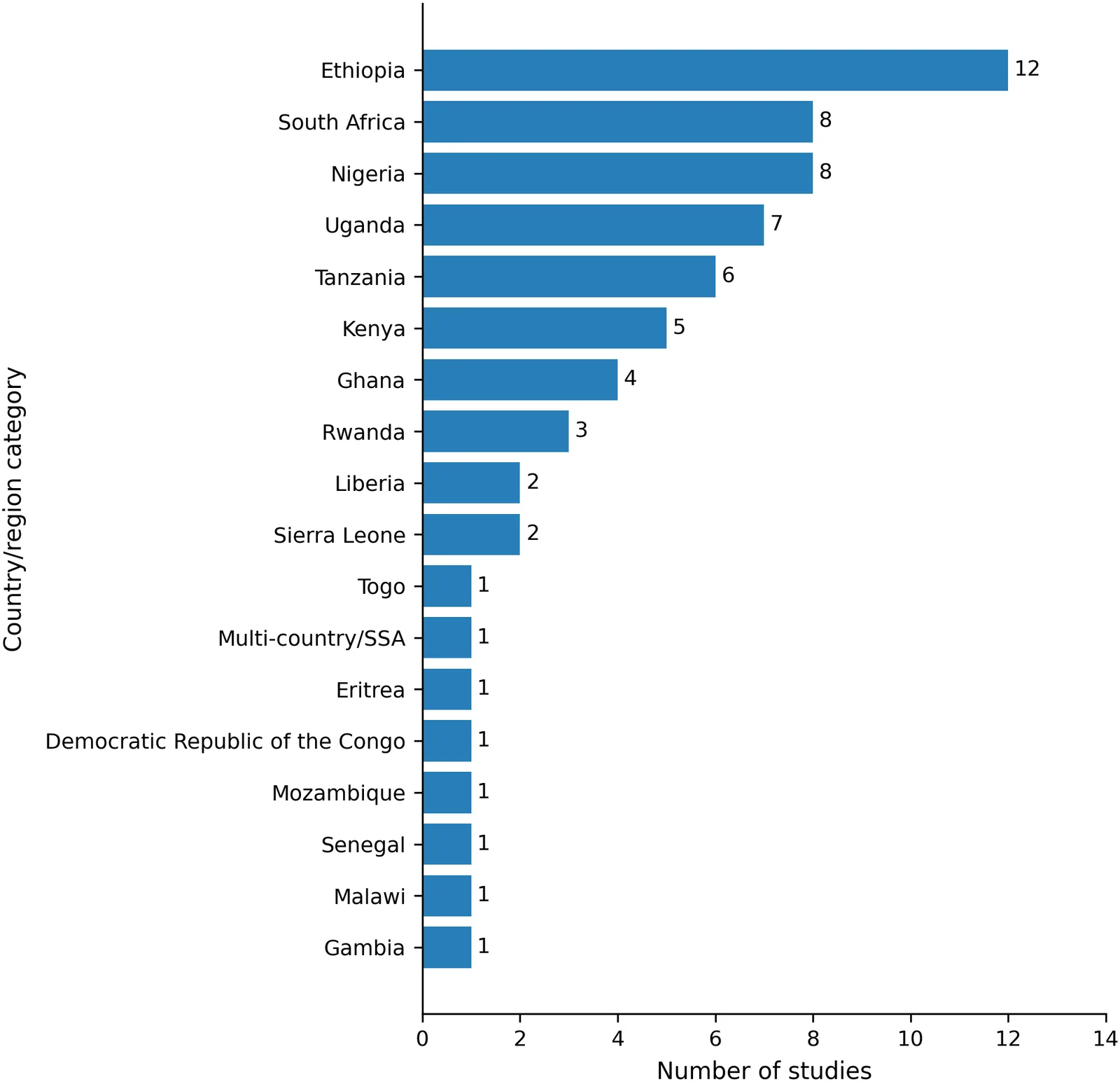
Geographical distribution of included studies across sub-Saharan Africa. Multi-country studies were counted once for each country represented; one pan-SSA conceptual study was classified as “Multi-country/SSA.”.

The included studies were conducted across several countries in SSA, although their geographical distribution was uneven (Fig 2). Ethiopia contributed the largest number of studies, followed by Nigeria, South Africa, Tanzania, Uganda, and Kenya. Collectively, these countries accounted for most of the available evidence, while relatively few studies were identified from other countries within the region. This distribution highlights the concentration of published evidence in a limited number of countries and indicates important geographical gaps in the literature.

The 64 included studies employed diverse methodological approaches, reflecting the breadth of research on strategies to improve outcomes of obstetric emergencies in SSA (Table 3). Cross-sectional studies constituted the largest proportion of the evidence base, followed by qualitative and cohort/observational studies. Quasi-experimental, intervention, pilot, and before-and-after studies were fewer in number, while randomized controlled trials, economic evaluations, implementation studies, interrupted time-series studies, and conceptual/framework papers were limited. Overall, the methodological profile indicates that the available evidence is largely descriptive, observational and implementation-oriented, with relatively few experimental studies evaluating intervention effectiveness.

**Table 3:** Methodological characteristics of the included studies (N = 64).

Cross-sectional studies	18	28.1
Qualitative studies	16	25.0
Cohort/observational studies	16	25.0
Quasi-experimental, intervention, pilot or before-and-after studies	4	6.3
Mixed-methods studies	3	4.7
Secondary data analyses	2	3.1
Implementation/programme evaluation study	1	1.6
Randomized controlled trial	1	1.6
Economic evaluation	1	1.6
Interrupted time-series study	1	1.6
Conceptual/framework paper	1	1.6
**Total**	**64**	**100.0**

### Thematic analysis

The included studies reported a wide range of strategies aimed at improving outcomes of obstetric emergencies in SSA. To facilitate synthesis and presentation, the identified strategies were organised into three broad domains: life-saving clinical and health-system interventions, health workforce education, training and mentorship, and health system organisation, referral mechanisms and policy implementation. Across all three domains, implementation was influenced by several contextual health-system and socio-environmental factors. An overview of the identified strategies is presented in Table 4.

**Table 4:** Overview of strategies identified across the included studies.

**Life-saving clinicalinterventions**	Pharmacological therapies	Magnesium sulfate, uterotonics, tranexamic acid, antibiotics
	Surgical interventions	Caesarean section, hysterectomy
	Critical care	HDUs, ICU care, blood transfusion
	Monitoring	Shock Index, MEOWS, lung ultrasound, maternal near-miss reviews
**Health workforce**	Education	Simulation training, EmONC training
	Mentorship	Clinical mentorship, CPD, coaching
	Task sharing	Associate clinicians, nurses, midwives
**Health system**	Referral systems	Ambulance services, MORES, referral coordination
	Service organisation	Decentralised CEmONC, obstetric HDUs
	Policies	Clinical protocols, MPDSR, NMCR, audit and feedback
**Contextual factors**	Health-system context	Infrastructure, staffing, equipment
	Community context	Delays, transport, cultural practices
	Resource context	Medicines, blood products, financing

### Theme 1: Life-saving interventions

The mapped evidence identified life-saving clinical and health-system interventions as the most frequently reported strategies for improving outcomes of obstetric emergencies in SSA. These interventions aimed to prevent maternal deterioration, reduce severe maternal morbidity, and improve survival through timely recognition, evidence-based clinical management, strengthened service delivery, and early escalation of care. As summarised in Table 5, the interventions were grouped into three interrelated categories: (i) clinical therapies, (ii) health-system strengthening interventions, and (iii) monitoring and triage approaches. [27,36,37,46,52].

**Table 5:** Life-saving interventions (n = 17).

Study	Aim	Maternal health outcomes
Geleto, Chojenta, 2020 [60]	Assess the incidence of maternal near misses and contributing factors among hospitals in Ethiopia. Assess the ability of hospitals to provide signal functions of emergency obstetric care and its regional distribution	Maternal near misses were unacceptably high, with significant regional variation. Recommendations include equitable resource distribution and quality improvement initiatives to address regional disparities.
Nathan, Seed, 2019 [36]	Evaluate shock index (SI) thresholds as predictors of outcomes in obstetric patients Women diagnosed with PPH	In hemorrhage, the risk of all outcomes increased with increasing “first” SI. In sepsis, the risk of all outcomes increased with increasing “worst”.
Heitkamp, Seinstra, 2019 [54]	Determine the incidence, risk indicators, and outcomes of emergency peripartum hysterectomy in Metro East, Cape Town, South Africa.	The incidence of EPH for sepsis was higher than previously reported. Repeat cesarean was strongly associated with EPH. Clinical characteristics of sepsis-related EPH compared unfavorably with those of hemorrhage-related EPH.
Rudakemwa, Cassidy, 2021 [42]	Assess reasons for ICU admission and accuracy of prediction models for mortality of obstetric patients.	There is a need for long-term follow-up, improved ICU capacity, validation of predictive tools, and adaptations for resource-limited settings.
Bekele, Fikre, 2022 [27]	Assess the factors affecting the utilization of antishock garments among obstetric care providers in public hospitals.	Utilization of antishock garments among obstetric care providers in Ethiopia, showing improved utilization and reduced maternal mortality with training and availability.
Unwaha, Bello, 2020 [31]	Assess the effectiveness of a 12- hour versus 24- hour intravenous maintenance dose of magnesium sulfate in women with pre- eclampsia, and the maternal and fetal outcomes.	A 12-hour maintenance dose of intravenous MgSO4 for managing severe pre-eclampsia is as effective and safe as the traditional 24-hour maintenance dose.
Yeshitila, Bante, 2021 [29]	Assess utilization of non-pneumatic anti-shock garment to control complications of post-partum hemorrhage and associated factors among obstetric care providers.	Application of NASG to women with obstetric hemorrhage. Adequate knowledge, training, and a positive attitude improved utilization of non-pneumatic anti-shock garment.
Prin, Kadyaudzu, 2019 [45]	Characterize ICU utilization for obstetric patients and its relationship with in-hospital mortality	ICU utilization for obstetric patients 23% of ICU admissions were obstetric. High in-hospital mortality rate of 49%. The majority required mechanical ventilation (95%) and vasopressors (48%).
Gonte, Peifer, 2023 [59]	Investigate the impact of the PPH Emergency Care package in a low-resource setting.	Implementation of PPH EmC was feasible and positively impacted PPH emergency care, (impacted facility and health system preparedness, referral coordination, teamwork, communication, and overall capacity to provide quality PPH emergency care).
Pisani, De Nicolo, 2020 [44]	Determine the frequency, timing, and type of pulmonary complications in critically ill obstetric patients.	Twenty-one percent had pulmonary complications. Complications were associated with poor outcomes.
Fagan et al., 2024 [68]	Assess impact of rural ambulance service on emergency obstetric care access.	Access to obstetric care rose from 18% to 66.5%; improved system integration.
Beza et al., 2024 [69]	Review admissions and outcomes in Ethiopia’s obstetric HDU.	1.4% maternal mortality; reduced ICU admissions; most cases managed in HDU.
Reynolds et al., 2024 [75]	Evaluate MORES system’s usability and impact on maternal referrals.	Improved referral communication and triage; reduced care delays.
Zewde et al., 2024 [76]	Assess WHO NMCR impact on maternal outcomes and care delays.	Reduced severe maternal outcomes and delays through structured NMCR implementation.
Abraha et al., 2025 [77]	To assess adverse birth outcomes and associated factors among mothers with antepartum hemorrhage	Maternal complications associated with antepartum hemorrhage; need for surgical intervention and escalation of care
Bulamba et al., 2025 [80]	To determine the incidence, risk factors, and outcomes of postpartum hemorrhage	Maternal mortality related to postpartum hemorrhage; treatment delays and escalation gaps
Teka et al., 2025 [86]	To determine the prevalence, clinical profile, and outcomes of obstructed labor	Maternal morbidity associated with obstructed labor; referral delays and surgical complications

#### Clinical therapies*.*

Clinical therapies primarily targeted the prevention and management of postpartum hemorrhage, hypertensive disorders of pregnancy, maternal sepsis, obstructed labor, and other life-threatening obstetric complications. Frequently reported interventions included non-pneumatic anti-shock garments (NASG), magnesium sulphate for severe pre-eclampsia and eclampsia, uterotonics, blood transfusion, emergency peripartum hysterectomy, and other evidence-based emergency obstetric treatments. Collectively, these interventions improved physiological stabilisation, reduced progression to severe complications, and supported timely management when implemented according to clinical guidelines by adequately trained healthcare providers.

Despite these benefits, several studies continued to report maternal morbidity and mortality, particularly where delays in diagnosis, limited availability of blood products, shortages of essential medicines, inadequate post-intervention monitoring, or delayed escalation of care constrained effective implementation. These findings suggest that the effectiveness of clinical therapies depends not only on the intervention itself but also on the capacity of the health system to deliver timely, high-quality care.

#### Systems-strengthening interventions*.*

Health-system strengthening interventions focused on improving the organisation, accessibility, and responsiveness of emergency obstetric care services. Commonly reported strategies included establishment of obstetric high-dependency units (HDUs), decentralisation of Comprehensive Emergency Obstetric and Newborn Care (CEmONC), strengthening referral coordination through initiatives such as the Mobile Obstetric Referral System (MORES), maternal near-miss and clinical review (NMCR) processes, rural ambulance services, and bundled emergency care packages for postpartum hemorrhage.[36,42,45,59,73–75].

These interventions generally enhanced referral efficiency, service readiness, continuity of care, and access to timely emergency obstetric services. While several studies reported improvements in severe maternal outcomes and health-system performance, others documented persistent ICU mortality, delays in referral, and resource limitations, indicating that health-system strengthening alone may be insufficient without concurrent improvements in workforce capacity and infrastructure.

#### Monitoring and triage tools.

Monitoring and triage approaches supported the early recognition of maternal deterioration and informed timely clinical decision-making during obstetric emergencies. Frequently reported approaches included obstetric early warning systems, shock index thresholds, lung ultrasound, maternal near-miss surveillance, ICU admission criteria, and assessments of Emergency Obstetric and Newborn Care (EmONC) signal functions.[77,80,86,87]. Although these approaches improved risk identification and prioritisation of care, their effectiveness depended on the availability of skilled personnel, functional referral systems, and adequate critical care capacity to enable timely escalation and definitive management.

Overall, the mapped evidence indicates that life-saving clinical and health-system interventions improve the recognition and management of obstetric emergencies and strengthen the delivery of emergency obstetric care. However, their effectiveness was consistently influenced by workforce capacity, referral efficiency, availability of essential resources, and broader health-system readiness, underscoring the importance of implementing these strategies as integrated components of emergency obstetric care rather than as isolated interventions.(Table 4).

### Theme 2: Health workforce education, training, and mentorship

Health workforce education, training, and mentorship emerged as key strategies for strengthening the management of obstetric emergencies across SSA. The mapped evidence demonstrated that capacity-building initiatives targeted healthcare providers’ knowledge, clinical competence, confidence, teamwork, and preparedness to recognise and manage life-threatening obstetric complications [33,37,39,40,46,47,57,63,88–90]As summarised in Table 6, these interventions included skills-and-drills training, competency-based Emergency Obstetric and Newborn Care (EmONC) education, pre-service and in-service training, continuing professional development (CPD), clinical mentorship, supportive supervision, and simulation- and digital learning approaches

**Table 6:** Health workforce education, training, and mentorship.

Study	Aim	Training gaps/ interventions
Thwala, Blaauw, 2019 [63]	Identify health system enablers and barriers to the delivery EmOC.	Lack of capacity of the health system to support the delivery of emergency obstetric care.
Okonofua, Ntoimo,2019 [40]	Assess the existing knowledge and skills relating to Emergency Obstetrics Care among health providers in eight referral maternity hospitals in Nigeria.	Participants knowledge and skills in offering specific EMOC services, and their confidence in transferring the skills to mid-level providers. Health providers scored less than 46% in a composite EMOC knowledge score. Doctors scored higher than the nurses/midwives.
**Nyamtema, LeBlanc, 2022 [37]**	Determine the causes and assess the factors that contributed to the maternal deaths based on the “three delays model.” Implemented capacity-building programs in emergency obstetric and newborn care in rural Tanzania.	The met need for emergency obstetric care increased significantly from 45% at baseline to 119% during the intervention period. The met need for emergency obstetric care in the control group also increased from 53% to 77%.
Kinnevey, Douglas,2021 [33]	Assess the effectiveness of a tailored down 5-day combined Helping Babies Survive (HBS)- Advanced Life Support in Obstetrics (ALSO) ALSO- A uterine balloon tamponade (UBT) course.	Training program for midwives in Uganda to teach the HBS curriculum HBS-ALSO-UBT course, resulting in improved self-assessment of confidence and knowledge in diagnosing and managing maternal and neonatal complications.
Rosenberg, Williams, 2021 [46]	Develop and implement a prehospital Emergency Obstetrics and Neonatal Course to improve maternal mortality in Rwanda.	Emergency Obstetrics and Neonatal Course, significantly improving participants’ scores and addressing gaps in care by adding critical medications to the supply chain
Nsangamay and Mash, 2019 [88]	Assess and improve the quality of care for women with PPH at Onandjokwe Hospital, Namibia through training of nursing and medical staff in obstetric emergencies, ensuring that guidelines and standard operating protocols were easily available.	Significant improvements were observed in meeting target standards, with all structural standards, six-ninth process standards, and two-thirds outcome standards achieved after intervention. Additionally, there was a reduction in maternal deaths, with one death in the baseline group and none in the follow-up group.
Mselle, O’Hearn, 2023 [89]	Explore how healthcare professionals, managers and community members experienced the implementation of a training program in comprehensive emergency obstetric and neonatal care training in rural Tanzania.	Increased skills and confidence in healthcare teams, improved community trust, and a reduction in maternal and neonatal mortality
Pattinson, Bergh, 2019 [47]	Determine whether there was a change in the number of maternal deaths and in the iMMR over time that could be attributed to the training of healthcare professionals with Off-site skills-and-drills training in EmOC.	Reductions of 29.3% in the number of maternal deaths and 17.5% in the number of maternal deaths from direct obstetric causes.
Germossa, Wondie,2022 [57]	Investigate the availability of CEmONC signal functions and describe lessons learned from Transform Health support (Clinical mentorship) in Developing Regional State in Ethiopia.	Availability of CEmONC signal functions improved, but maternal and neonatal mortality remained unchanged, indicating a need for further investigation and quality assessments.
Gakwerere et al., 2024 [71]	Assess uptake and accessibility of midwives and nurses to CPD and determine their knowledge and skills gaps in key competencies of EmONC to inform the CPD programming. CPD workshops and mentorship were implemented; the 60-credit requirement for licensure was enforced, and knowledge levels were assessed.	79.9% failed to meet CPD requirements; significant gaps in EmONC knowledge noted; mentorship showed potential.
Shikuku et al., 2024 [72]	Measure the effectiveness of pre-service Emergency obstetric care training intervention package on the knowledge and skills of final year midwifery students in Kenya. App-based videos and simulation-based curriculum introduced with faculty mentorship and structured teaching.	Educator skills and student knowledge improved; resource and faculty shortages have a limited impact.
Shannon et al., 2024 [73]	Assess patient awareness of DS, identify barriers to awareness, and evaluate potential for implementing smartphone-based technologies for education.	Actual knowledge was low despite high self-perceived awareness; strong interest in digital health education.
Govender et al., 2025 [81]	To assess emergency care providers’ knowledge, attitudes, and practices related to obstetric hemorrhage management	Provider preparedness influencing the timely recognition and management of obstetric hemorrhage
Lunda et al., 2025 [83]	To determine maternal mortality ratios and avoidable factors before and during the COVID-19 period	Maternal deaths, provider- and system-related avoidable factors contributing to mortality

#### Workforce education and competency development.

Across the included studies, education and competency-based training consistently improved providers’ knowledge, technical skills, confidence, and adherence to evidence-based clinical guidelines for managing obstetric emergencies [33,37,39,40,46,47,57,63,88–90]. Training interventions targeted healthcare providers’ knowledge, skills, and confidence in managing obstetric emergencies and included skills-and-drills courses, mentorship programs, pre-service and in-service training, continuous professional development (CPD), and emerging digital and simulation-based approaches (Table 6). Skills-and-drills training, simulation-based education, structured EmONC courses, and competency-focused learning enhanced providers’ ability to recognise obstetric complications, make timely clinical decisions, and work effectively within multidisciplinary teams. Several studies also reported improvements in selected process indicators, including availability of EmONC signal functions, provider preparedness, and service readiness following implementation of these educational interventions [81,83].

#### Mentorship and continuing professional development.

Clinical mentorship and continuing professional development complemented formal training by reinforcing knowledge and supporting the translation of skills into routine clinical practice. Mentorship programmes, supportive supervision, structured feedback, and CPD initiatives strengthened adherence to clinical protocols and promoted continuous professional learning, although important competency gaps persisted in several settings [58,72]. One study reported that nearly 80% of providers had not met CPD requirements despite implementation of mentorship programmes, highlighting the need for sustained investment in workforce development [72].

#### Digital learning and innovative educational approaches.

Emerging digital and technology-assisted educational strategies were also identified. Smartphone applications, app-based videos, simulation-supported curricula, and digital learning resources improved educators’ capacity, students’ knowledge, and providers’ engagement while demonstrating potential to expand access to emergency obstetric education in resource-constrained settings [73,74]. However, shortages of trained faculty, educational resources, and infrastructure continued to limit implementation and scalability of these approaches.

Although education, training, and mentorship interventions consistently strengthened provider competence and improved several process outcomes, their effects on maternal mortality were less consistent. Some studies demonstrated improvements in service readiness and availability of EmONC signal functions without corresponding reductions in maternal or neonatal mortality, while others reported persistent gaps in provider knowledge, preparedness, and timely recognition of obstetric emergencies [58,72,83,85]. Overall, the mapped evidence suggests that workforce strengthening is an essential component of improving obstetric emergency care but is most effective when implemented alongside broader health-system strengthening strategies.

### Theme 3: Health system organisation, referral mechanisms, and policy implementation

Health system organisation, referral mechanisms, and policy implementation constituted a major domain of strategies identified to improve outcomes of obstetric emergencies in SSA. The mapped evidence demonstrated that these strategies focused on strengthening the organisation, governance, accessibility, and responsiveness of emergency obstetric care across different levels of the health system As summarised in Table 7, interventions included strengthening Emergency Obstetric and Newborn Care (EmONC) services, decentralising comprehensive emergency obstetric care, improving referral and transport systems, implementing clinical policies and protocols, and institutionalising quality improvement and maternal surveillance mechanisms.

**Table 7:** Health System organization, Referral Mechanisms and Policy Implementation (n = 13).

Study	Aim	Health system organization/ referral mechanisms/ policy
Cavallaro, Benova,2020 [61]	Establish where women deliver in Senegal and how this has changed.	Lack of timely access to care due to distance to a CEmOC facility, need to increase the capacity of rural centers where most births occur.
Mpunga Mukendi, Chenge, 2019 [38]	Assess emergency obstetric care’s availability, quality, and equity in the DRC.	The distribution and quality of EmOC are problematic. Lack of regulation and monitoring is a crucial factor. Recommendations include establishing and monitoring appropriate standard operating procedures for providers.
Mukuru, Kiwanuka, 2021 [48]	Explore the barriers frontline implementers of EmOC policies faced, their coping behaviors, and the consequences for maternal health.	Policy and implementation system incompatibility, inadequate inputs, supplies, workforce, and skills hindered the implementation of EmOC. This led to unresponsive EmOC services and delayed interventions for mothers at different care levels.
King, Tarway-Twalla, 2022 [34]	Assess the quality of childbirth care in Liberia, focusing on readiness for emergency care and referral, staffing, and the volume of births in health facilities All health facilities in Liberia.	Although facility births increased from 37% to 80% between 2004 and 2017, only 18% of facilities could perform basic emergency obstetric and neonatal care (EmONC), and just 8% offered blood transfusions and cesarean sections. Additionally, 63% of births occurred in facilities lacking full emergency readiness, and 60% of facilities could not make emergency referrals.
Banke-Thomas, Wong, 2021 [24]	Derive and compare estimates of travel time to reach comprehensive emergency obstetric care (CEmOC).	Significant gaps in geographical access to life-saving services like comprehensive emergency obstetric care (CEmOC).
**Dominico, Serbanescu, 2022 [91]**	Evaluate the outcome and impact of the comprehensive approach to improving Emergency Obstetric and Newborn Care program.	Decentralizing comprehensive EmONC and training associate clinicians and nurses resulted in noticeable enhancements in the accessibility and use of life-saving birth care in Kigoma. Leading to reduce MMR.
Kabo, Orobaton, 2019 [55]	Evaluate the impact of quality improvement interventions for obstetric emergencies following a baseline assessment. Twenty-one hospitals are designated as comprehensive EmOC centers.	The rise in facility-based births and the need for EmOC suggests increased availability, access, and utilization of EmOC.
Jejaw, Debie, 2021 [56]	Evaluate the implementation of a comprehensive emergency obstetric and newborn care program in terms of availability, compliance, and acceptability dimensions at the University of Gondar Comprehensive Specialized Hospital, Northwest Ethiopia.	The implementation status of the CEmONC program was good compliance with guidelines needed improvement. Essential emergency drugs and equipment were often stocked out.
Aikpitanyi, Ohenhen, 2019 [52]	Identify the medical causes and contributory factors of maternal mortality through the MPDSR process and elucidate the policy responses following the dissemination of the results.	Establishment of Maternal and Perinatal Death Surveillance and Response Committee. Improved quality of care and a significant decline in the maternal mortality ratio at the referral hospital.
Abdullahi et al., 2025 [78]	To compare maternal near-miss incidence and outcomes between secondary and tertiary facilities	Maternal near-miss incidence; maternal mortality; mortality index across levels of care
Tarimo et al., 2025 [79]	To examine barriers to skilled birth attendance and emergency obstetric care	Delayed access to emergency obstetric care associated with adverse maternal outcomes
Keem et al., 2025 [84]	To explore women’s experiences of referral following obstetric emergencies	Referral-related delays affecting timely access to definitive care
Cooper et al., 2025 [85]	To assess whether antenatal risk stratification predicts adverse obstetric outcomes	Maternal complications occurring despite low-risk antenatal classification

#### Health system organisation and service delivery.

Several studies reported strategies aimed at strengthening the organisation and delivery of emergency obstetric care through expansion of Comprehensive Emergency Obstetric and Newborn Care (CEmONC) services, decentralisation of specialist care, establishment of functional referral networks, and improvements in facility readiness [24,34,38,48,56,61].

#### Referral mechanisms and emergency transport systems.

Strengthening referral pathways and emergency transport systems was consistently identified as a strategy for improving timely access to definitive obstetric care [53,56,76,80–82]. Reported interventions included structured referral coordination systems, rural ambulance services, the Mobile Obstetric Referral System (MORES), referral communication platforms, and standardised referral protocols. Collectively, these approaches improved communication between facilities, reduced referral delays, enhanced continuity of care, and facilitated more timely management of women experiencing obstetric emergencies. Nevertheless, persistent delays related to transport, geographical accessibility, and coordination continued to affect referral efficiency in several settings [81,82,86,87].

#### Policy implementation, governance, and quality improvement.

Policy implementation and governance strategies focused on strengthening the quality and accountability of emergency obstetric care through implementation of national clinical guidelines, Emergency Obstetric and Newborn Care policies, maternal and perinatal death surveillance and response (MPDSR), WHO maternal near-miss and clinical review (NMCR) processes, clinical audit, and quality improvement initiatives [30,53,56,57,62,77]. These strategies supported standardisation of clinical practice, improved monitoring of maternal outcomes, strengthened accountability, and facilitated identification of avoidable factors contributing to maternal morbidity and mortality. Several studies reported improvements in access to emergency obstetric services and selected maternal outcomes following implementation of these system-level reforms, although effectiveness varied according to local health-system capacity and implementation fidelity.

Overall, the mapped evidence indicates that strengthening health system organisation, referral mechanisms, and policy implementation enhances the accessibility, coordination, and quality of emergency obstetric care. However, the effectiveness of these strategies depended on adequate infrastructure, functional referral systems, sufficient human resources, leadership, governance, and sustained implementation across all levels of the health system [24,34,38,48,56,61].

### Additional findings: Contextual factors influencing the implementation of strategies

Although not identified as strategies within the scope of this review, the included studies consistently reported contextual factors that influenced the implementation and effectiveness of interventions aimed at improving outcomes of obstetric emergencies. These factors were identified across all thematic areas and affected the delivery, uptake, sustainability, and impact of clinical, workforce, and health-system strategies.[23,25,26,30,35,41,49–51,53,58,62,64–67]. As summarised in Table 8, the contextual factors clustered into four interrelated domains: health-system capacity, workforce constraints, resource availability, and delays in accessing and receiving care.

**Table 8:** Contextual factors influencing the implementation of strategies to improve outcomes of obstetric emergencies in SSA (n = 21).

Study	Aim	Contextual/ System barriers
Sevene, Boene, 2021 [30]	Describe the feasibility of task-sharing, the initial screening and initiation of obstetric emergency care for pre-eclampsia/eclampsia.	Task-sharing for screening and pre-referral management of pre-eclampsia and eclampsia was considered viable and well-received at the community level. However, it is important to address the challenges at the health system level to reduce MMR.
Kaselitz, James, 2019 [35]	Explore basic and comprehensive emergency obstetric service provision across four districts in rural northern Ghana, and whether women were more likely to deliver at facilities with more skilled care.	Provision of basic and comprehensive emergency obstetric services in Ghana, highlighting the availability and performance of facilities at different health levels.
Marotta, Di Gennaro, 2020 [32]	Explore the value-based dimension by performing a cost-utility analysis concerning the implementation and one-year operation of the HDU. (quality-adjusted life-years) of patients admitted to the HDU during the study period.	In resource-limited settings, obstetric HDUs can be regarded as a highly cost-effective and efficient innovation.
Daniels and Abuosi, 2020 [23]	Identify barriers to referring emergency obstetric cases to the leading national referral center.	Barriers included communication barriers, referral transportation systems, health infrastructure, supplies, and human resource constraints. Implications on type II and III of the three delays model.
Nabulo, Gottfredsdottir, 2023 [26]	Explore the health system and client-related factors to understand how these could have facilitated or hindered the referral process.	The experience of obstetric referral for women was unpleasant due to delays and inadequate quality of care, which contributed to perinatal mortality and maternal morbidities. Training healthcare providers in respectful maternity care (RMC) could enhance care quality.
Ameyaw, Amoah, 2020 [65]	Assess the resources available for managing comprehensive emergency obstetric and neonatal care and referral services Maternity facilities in ten hospitals.	Limited human resources and equipment severely hamper the management of emergency obstetric care and referrals in Northern Ghana.
Ayawine and Atinga, 2022 [66]	Explore user and community responses to service delivery gaps in emergency obstetric care provision in rural Ghana.	Gaps in service delivery impact on maternal health outcomes.
Alobo, Ochola, 2021 [53]	Explore and describe the pathways for women requiring emergency obstetrics and newborn care and understand delays in accessing care after reaching a health facility.	Pathways to emergency obstetric and newborn care, causes of delays in care, and the impact on maternal and neonatal outcomes.
Geleto, Chojenta, 2020 [60]	Investigate the perceptions of midwives about the quality of emergency obstetric care provided at hospitals in the Harari region of Ethiopia.	Several interdependent factors limited the quality of emergency obstetric care including inadequate number of midwives, scarce training opportunities, language barriers, frequent disruptions to medical supplies, lack of treatment protocols, poor supportive supervision, and poor staff motivation. Recommendations for quality improvement initiatives, equitable resource distribution, and strengthening existing health infrastructure, resources, and service delivery management.
Dougherty, Gobezayehu, 2023 [58]	Determine Amhara’s clinical readiness and quantify the relationship between SF (signal function) and CC (clinical cascade) estimates of preparedness to manage obstetric emergencies.	Facilities unprepared to manage common obstetric emergencies.
Mwaniki, Edwards, 2020 [49]	Evaluate the completeness of the maternal death review (MDR) process, focusing on aspects such as notification, audit completion, documentation quality, causes of maternal deaths, hospital-related delays, missed opportunities, and action points in seven hospitals within Thika sub-county, Kenya.	Community-based maternal death reporting and reviews are not routinely done, thus contributing to the overall underreporting.
Akter, Forbes, 2022 [50]	Explore healthcare providers’ knowledge and practices of PPH detection and management after vaginal birth in nine hospitals in Kenya, Nigeria, and South Africa	Lack of expertise in PPH detection and reliance on visual estimation can delay PPH management. Common barriers include staff shortages, insufficient resources, and late inter-hospital referrals.
Mislu, Seid, 2023 [64]	Assess the magnitude of maternal third delay and associated factors among women admitted for emergency obstetric care.	Delayed access to emergency obstetric care
Ramavhoya, Maputle, 2020 [41]	Explore and describe the experiences of midwives in managing women diagnosed with hypertensive disorders during pregnancy in rural areas.	High Maternal Mortality Rate and influencing factors like inadequate quality care, inadequate transport, and lack of skills.
Moore, Thomson, 2019 [25]	Introduce a modified obstetric early warning system and assess its feasibility and potential impact on improving postoperative monitoring and earlier identification and management of deteriorating patients.	Modified obstetric early warning system in Ethiopia, which led to earlier identification and management of deteriorating postoperative patients.
Teka, Yemane, 2022 [62]	Examine the prevalence of maternal morbidities and deaths.	A significant mortality contributor was a lack of ICU admission for pregnant women with severe maternal complications: sepsis, hemorrhage.
Anto-Ocrah, Cushman, 2022 [51]	Propose an access framework that integrates the Three Delays Model in maternal health with emergency care (EC) interventions.	Improved timely access to emergency obstetric care and reduction in maternal mortality in SSA.
Adebayo et al., 2024 [70]	Determine the prevalence of primary postpartum haemorrhage (PPH), risk factors, and maternal and neonatal outcomes in a multi-centre study across Nigeria. Used uterotonics for PPH management; enhanced skilled care and hospital data systems for monitoring outcomes.	PPH affected 3.2% of women; 4.8% of maternal deaths occurred among PPH cases; the stillbirth rate was 25%; gaps in care persisted.
Birabwa et al., 2024 [74]	Investigate care-seeking delays and referral pathways in urban Kampala facilities.	Multiple delays in reaching care, stillbirths, and inefficiencies in the referral process were reported.
Nyamtema, Mtey,2021 [92]	Determine the causes and assess the factors that contributed to the maternal deaths based on the “three delays model.” Training intervention: trained in CEmONC and anesthesia.	The met need for emergency obstetric care increased significantly from 45% at baseline to 119% during the intervention period. The met need for emergency obstetric care in the control group also increased from 53% to 77%.
Sengoka et al., 2025 [82]	To explore the lived experiences of women who survived severe maternal complications	Long-term physical, psychological, and social consequences following obstetric emergencies

#### Health-system capacity.

Health-system limitations were the most frequently reported contextual factors influencing implementation of obstetric emergency strategies. Studies described inadequate health infrastructure, limited critical care capacity, fragmented referral systems, weak governance, and deficiencies in health information systems that constrained timely and effective emergency obstetric care [24,26,27,33,36,42,50–52,54,59]. These limitations reduced service readiness and restricted the ability of health facilities to consistently deliver evidence-based emergency obstetric interventions despite the availability of recommended clinical and health-system strategies..

#### Workforce constraints.

Workforce-related challenges included shortages of skilled healthcare providers, inadequate supervision, high staff turnover, uneven distribution of trained personnel, and limited opportunities for continuing professional development [31,54,59,63,65–67,84]. These factors affected providers’ ability to maintain competency, recognise maternal deterioration promptly, adhere to evidence-based protocols, and sustain improvements achieved through education, mentorship, and health workforce strengthening initiatives.

#### Resource availability.

Resource constraints were reported throughout the evidence base and included shortages of essential medicines, blood products, equipment, transport, and financial resources required to support emergency obstetric care [35,39,49,57,62,82]. Limited availability of these resources frequently reduced the effectiveness of otherwise evidence-based clinical and health-system interventions, highlighting the importance of adequate health-system investment for successful implementation.

#### Delays across the continuum of care.

Several studies also highlighted persistent delays in recognising obstetric complications, deciding to seek care, reaching appropriate health facilities, and receiving definitive treatment after arrival [24,27,50,59,75,93]. These delays reflected broader geographical, organisational, socioeconomic, and health-system challenges that continued to undermine the timely delivery of emergency obstetric care despite implementation of evidence-based strategies.

Collectively, these findings indicate that contextual factors influence the implementation and effectiveness of strategies across all domains of obstetric emergency care. Although these factors were not the primary focus of this review, they consistently shaped how interventions were delivered, adopted, and sustained, emphasising the importance of considering local health-system context when implementing strategies to improve maternal outcomes in SSA

## Discussion

This scoping review mapped strategies reported to improve outcomes of obstetric emergencies in SSA and demonstrated that effective emergency obstetric care requires coordinated interventions across clinical, workforce, and health-system domains. Rather than identifying a single intervention sufficient to improve maternal outcomes, the mapped evidence showed that successful management of obstetric emergencies depends on the integration of evidence-based clinical care, competent and supported health workers, functional referral systems, responsive service organisation, and enabling implementation contexts. This finding aligns with global maternal health guidance emphasizing that reductions in maternal morbidity and mortality require not only increased service coverage, but also improvements in quality of care, health-system readiness, timely referral, and emergency response capacity [15].

### Life-saving interventions

This review identified life-saving clinical and health-system interventions as the cornerstone of strategies reported to improve outcomes of obstetric emergencies in SSA. Across the included studies, interventions aimed at strengthening emergency obstetric care contributed to earlier recognition of maternal deterioration, improved adherence to evidence-based clinical management, enhanced service readiness, and better coordination of emergency care [28,30,32,37,43,45,46,55,60,61,69,70,76–78,82,88]. Collectively, these findings demonstrate that improving maternal outcomes depends not only on timely clinical management but also on the capacity of health systems to deliver life-saving interventions rapidly and consistently.

These findings are consistent with the World Health Organization’s recommendations on Emergency Obstetric and Newborn Care (EmONC) and Essential Emergency and Critical Care (EECC), which emphasise that high-quality emergency obstetric care requires coordinated clinical management supported by functional referral pathways, appropriate monitoring, and health-system readiness rather than isolated therapeutic interventions [93,94]. Similarly, recent global evidence indicates that improvements in maternal survival are achieved when evidence-based clinical interventions are implemented within health systems that ensure timely access to comprehensive emergency obstetric services, adequate infrastructure, and multidisciplinary care [95,96].

A significant contribution of this review is the demonstration that clinical interventions alone are insufficient to achieve sustained improvements in maternal outcomes. Across the mapped evidence, interventions were most successful when accompanied by health-system strengthening measures such as improved referral pathways, enhanced critical care capacity, standardised monitoring systems, and quality improvement initiatives [37,43,45,46,55,60,61,69,70,76–78,82,88]. Conversely, where these enabling systems were weak, improvements in clinical processes did not consistently translate into reductions in maternal mortality or severe maternal outcomes. This finding reinforces the growing consensus that effective obstetric emergency care requires coordinated implementation of both clinical and health-system interventions rather than isolated therapeutic approaches.

These findings highlight the importance of implementing life-saving clinical interventions as part of integrated emergency obstetric care systems rather than as isolated clinical practices. Strengthening referral pathways, ensuring uninterrupted availability of essential medicines and blood products, expanding critical care capacity, and embedding continuous quality improvement within maternity services are likely to maximise the effectiveness and sustainability of evidence-based interventions aimed at reducing maternal morbidity and mortality in SSA.

### Health workforce education, training, and mentorship

This review identified health workforce education, training, and mentorship as fundamental strategies for strengthening the management of obstetric emergencies across SSA. The mapped evidence demonstrated that competency-based education, simulation training, clinical mentorship, continuing professional development (CPD), and digital learning approaches consistently improved healthcare providers’ knowledge, technical competence, confidence, teamwork, and adherence to evidence-based clinical protocols [34,38,40,41,47,48,58,64,72–74,83,85,89,90]. These findings highlight the central role of a competent and well-supported health workforce in improving the quality and responsiveness of emergency obstetric care.

Health workforce training and mentorship initiatives were widely implemented to strengthen provider competence in recognising and managing obstetric emergencies, particularly among midwives and other frontline cadres. Consistent with existing systematic reviews, training interventions were associated with improvements in provider knowledge, skills, and adherence to clinical protocols [16,97]. Simulation-based education, in particular, has demonstrated value in improving clinical decision-making and team performance in obstetric emergencies, especially in settings with limited exposure to high-acuity cases [16,19]. Continuing professional development (CPD) approaches were also highlighted as important mechanisms for sustaining competence over time. Recent evidence indicates that CPD programmes for midwives can support improved management of obstetric complications, although their effectiveness varies depending on implementation quality and system support [18]. Despite these gains, this review found that training interventions demonstrated variable effects on maternal mortality, reflecting a well-documented challenge in translating individual-level capacity building into sustained clinical outcomes [98]. Training benefits were frequently undermined by workforce shortages, high workload, limited supervision, and a lack of essential resources. These findings support the view that workforce development strategies are most effective when embedded within broader organisational reforms that promote supportive supervision, adequate staffing, and enabling work environments.

### Health System organization, Referral Mechanisms and Policy Implementation

This review identified health system organisation, referral mechanisms, and policy implementation as essential strategies for improving outcomes of obstetric emergencies in SSA. The mapped evidence demonstrated that strengthening Emergency Obstetric and Newborn Care (EmONC) services, decentralising comprehensive emergency obstetric care, improving referral coordination, strengthening emergency transport systems, implementing evidence-based clinical protocols, and institutionalising quality improvement and maternal surveillance mechanisms enhanced access to timely emergency obstetric care and improved service delivery across diverse healthcare settings [25,30,35,39,49,53,56,57,62,80,81,86,87].

These findings highlight the importance of well-organised and responsive health systems in supporting effective management of obstetric emergencies.The organization of emergency obstetric care and the functionality of referral mechanisms emerged as critical determinants of timely access to life-saving interventions. Studies of emergency obstetric care policies and referral systems consistently identified gaps between policy intent and effective implementation, particularly in peripheral and rural facilities [23,95]. Although facility-based deliveries have increased across many settings, this expansion has not consistently translated into improved outcomes where facilities lack basic or comprehensive emergency obstetric readiness.

Strengthening referral coordination, decentralising comprehensive emergency obstetric and newborn care services, and implementing clear standard operating procedures were associated with improved service utilisation and, in some contexts, reductions in maternal mortality. These findings are consistent with evidence demonstrating that geographic proximity to functional emergency care and efficient referral pathways are essential for survival during obstetric emergencies [51,95]. However, persistent barriers related to transport availability, communication breakdowns, and inter-facility coordination continue to contribute to avoidable delays and adverse outcomes. Weak accountability and monitoring mechanisms further limit the effectiveness of emergency obstetric care policies, reinforcing the importance of linking policy implementation with robust measurement and learning systems [96].

### Additional findings: Contextual factors influencing the implementation of strategies

An important finding of this review was the consistent influence of contextual factors on the implementation and effectiveness of strategies to improve outcomes in obstetric emergencies across SSA. Across the included studies, health-system limitations, workforce shortages, inadequate infrastructure, stock-outs of essential medicines and blood products, limited critical care capacity, and weaknesses in referral systems frequently constrained the implementation and sustainability of life-saving clinical, workforce, and health-system interventions [24,26,27,31,33,36,42,50–52,54,59,61,63,65–67,71,75,84,93]. These findings demonstrate that the effectiveness of evidence-based interventions is strongly shaped by the health-system context in which they are implemented.

The contextual factors identified in this review align with the broader maternal health literature, which recognizes health-system readiness as a critical determinant of maternal survival in low-resource settings [99]. Persistent shortages of skilled health workers, inadequate infrastructure, limited access to emergency transport and critical care services, and interruptions in the availability of essential medicines and blood products continue to undermine the delivery of timely, high-quality emergency obstetric care, despite increasing coverage of facility-based births and emergency obstetric services.

The findings also reinforce the continued relevance of the Three Delays Model as a framework for understanding persistent maternal morbidity and mortality [100]. Similar to previous studies conducted in SSA and other low-resource settings, this review found that delays in recognising complications, reaching appropriate health facilities, and receiving definitive treatment were frequently driven by transport challenges, inefficient referral pathways, limited facility readiness, and organisational constraints [99,101]. These interconnected delays often reduced the effectiveness of otherwise evidence-based clinical and health-system interventions.

The findings highlight that sustainable improvements in obstetric emergency care require implementation strategies responsive to local health-system contexts. Investments in clinical interventions, workforce development, and health-system reforms should therefore be paired with strengthened governance, referral coordination, infrastructure, supply chains, health information systems, and continuous quality improvement to ensure that evidence-based strategies can be implemented effectively and sustained over time.

## Strengths and limitations

This review has several limitations. First, the search was restricted to studies published in English between January 2019 and December 2025; consequently, relevant studies published in other languages or outside this time frame may not have been captured. Second, although a comprehensive search was conducted across multiple electronic databases and supplemented by manual reference list screening, unpublished studies, grey literature not indexed in the selected databases, and ongoing implementation initiatives may have been missed. Third, the included studies were methodologically heterogeneous, encompassing qualitative, quantitative, mixed-methods, implementation, and conceptual studies conducted across diverse healthcare settings. This heterogeneity precluded formal comparison of intervention effectiveness or quantitative synthesis and is consistent with the exploratory nature of a scoping review. Fourth, although methodological quality appraisal was undertaken to support interpretation of the evidence, quality assessments were not used to exclude studies, in keeping with established scoping review methodology. Consequently, the review maps the breadth of available evidence rather than the effectiveness of specific interventions.

Despite these limitations, this review has several important strengths. It provides a comprehensive and up-to-date synthesis of evidence published between 2019 and 2025, maps the full spectrum of strategies used to improve outcomes of obstetric emergencies across SSA, and integrates clinical, workforce, health-system, and implementation perspectives within a single review. By identifying both evidence gaps and contextual factors influencing implementation, the review offers a broad evidence base to inform future research, policy, and implementation of integrated strategies to strengthen emergency obstetric care in resource-constrained settings.

## Conclusion and recommendations

### Conclusion

This scoping review mapped the range of strategies reported to improve outcomes of obstetric emergencies in SSA and demonstrated that effective emergency obstetric care depends on integrated, context-responsive approaches rather than isolated interventions. The evidence indicates that improvements in maternal outcomes require coordinated implementation of life-saving clinical interventions, a competent and well-supported health workforce, functional referral systems, responsive health service organisation, and effective policy implementation within adequately resourced health systems. The review further highlights that contextual factors—including workforce shortages, infrastructure limitations, resource constraints, and delays in accessing and receiving care—strongly influence the implementation and effectiveness of these strategies.

The findings emphasize the importance of strengthening health systems alongside evidence-based clinical care to achieve sustainable reductions in maternal morbidity and mortality. Policymakers, programme implementers, and healthcare leaders should prioritise integrated strategies that combine clinical excellence with workforce development, strengthened referral systems, quality improvement, and robust governance to improve emergency obstetric care across diverse settings in SSA.

### Implications for policy, practice, and research

#### Implications for policy.

The findings of this review suggest that policies aimed at reducing maternal morbidity and mortality should move beyond isolated clinical interventions towards integrated health-system strengthening. Policymakers should prioritize investments that simultaneously strengthen Emergency Obstetric and Newborn Care (EmONC) services, referral and transport systems, health workforce capacity, critical care services, supply chains for essential medicines and blood products, and maternal surveillance systems. National maternal health policies should also emphasize effective implementation, monitoring, and continuous quality improvement to ensure that evidence-based interventions are translated into routine practice across all levels of the health system.

#### Implications for practice.

For healthcare providers and health service managers, the review highlights the importance of delivering evidence-based obstetric emergency care within well-functioning systems of care. Clinical interventions should be supported by regular competency-based education, simulation training, structured mentorship, continuing professional development, multidisciplinary teamwork, standardised clinical protocols, and effective referral coordination. Healthcare facilities should also strengthen early recognition of maternal deterioration, improve communication across referral networks, and institutionalise routine audit and maternal death surveillance processes to promote learning and accountability.

#### Implications for research.

The review identified important evidence gaps that warrant further investigation. Future research should evaluate integrated packages of clinical, workforce, and health-system interventions rather than individual strategies in isolation. Greater emphasis should be placed on implementation research that examines how interventions are adapted, implemented, scaled, and sustained across diverse resource-constrained settings. There is also a need for more rigorous prospective and experimental studies evaluating long-term maternal and neonatal outcomes, implementation fidelity, cost-effectiveness, and health-system impacts. Furthermore, research from underrepresented countries within SSA is needed to improve the geographical representativeness of the evidence base and to inform context-specific policy and practice.

### Recommendations

Based on the mapped evidence, the following recommendations are proposed to strengthen the prevention and management of obstetric emergencies and improve maternal outcomes in SSA.

1 **Strengthen integrated emergency obstetric care**

Health systems should prioritize implementing integrated emergency obstetric care that combines evidence-based clinical interventions with functional referral systems, timely access to Comprehensive Emergency Obstetric and Newborn Care (CEmONC), critical care services, and effective monitoring and triage. Investments should focus on ensuring uninterrupted availability of essential medicines, blood products, equipment, and appropriately staffed emergency obstetric services to optimize timely management of life-threatening complications.

2 **Invest in sustainable health workforce development**

Governments, professional regulatory bodies, and training institutions should institutionalize competency-based pre-service and in-service education, structured continuing professional development (CPD), simulation-based training, clinical mentorship, and supportive supervision for healthcare providers involved in emergency obstetric care. These initiatives should be embedded within comprehensive workforce strategies that address staffing shortages, retention, equitable deployment, workload management, and career development to ensure sustained improvements in quality of care.

3 **Strengthen health system organisation, referral systems, and governance**

Health system leaders should strengthen the organisation and governance of emergency obstetric care by improving referral coordination, expanding equitable access to functional EmONC and CEmONC services, decentralizing emergency obstetric care where appropriate, implementing evidence-based clinical guidelines and standard operating procedures, and institutionalizing maternal and perinatal death surveillance and response (MPDSR) together with continuous quality improvement initiatives. Effective implementation should be supported by strong leadership, accountability mechanisms, routine monitoring, and use of health information for decision-making.

4 **Address implementation context and health-system constraints**

Implementation of obstetric emergency strategies should explicitly address contextual factors that influence their effectiveness. Policymakers and program implementers should prioritize investments in health infrastructure, emergency transport, supply chain management, digital health systems, communication networks, and financing mechanisms that improve service readiness and reduce delays across the continuum of care. Tailoring implementation strategies to local health-system contexts will enhance adoption, sustainability, and long-term impact.

5 **Prioritize implementation research and evaluation**

Future research should move beyond evaluating individual interventions towards implementation research examining integrated packages of clinical, workforce, and health-system strategies. Well-designed prospective, mixed-methods, and implementation studies are needed to evaluate effectiveness, implementation fidelity, scalability, sustainability, cost-effectiveness, and long-term maternal and neonatal outcomes across diverse settings in SSA. Attention should be given to underrepresented countries and health-system contexts to strengthen the regional evidence base and support context-specific policy and practice.

## Supporting information

S1 File Search strategies used for the electronic databases. (DOCX)

S1 Checklist PRISMA extension for Scoping Reviews (PRISMA-ScR) checklist. (DOCX)
