# Supplementary material for: Strategies to improve outcomes of obstetric emergencies in sub-Saharan Africa: A scoping review
Source: PLoS One. 2026 Sep 1;21(9):e0355174. doi: 10.1371/journal.pone.0355174 (PMC13533365; doi:10.1371/journal.pone.0355174)
Supplement: Supplementary file 1 [file pone.0355174.s003.pdf]

Critical care interventions to improve the outcome of obstetric emergencies in sub-Saharan Africa: A scoping review

Manuscript Number: PONE-D-25-31834

Main claims of the paper and its significance for the discipline?

The authors aimed to identify critical care strategies to improve outcomes of obstetric emergencies in sub-Saharan Africa and to strengthen maternal health systems. However, the content of the article does not fully reflect the title or stated objectives. Based on the title, one would expect a focus on clinical care interventions aimed at improving emergency obstetric conditions, as alluded to in Lines 109-110 in the introduction. To align the outcomes more closely with the title, a scoping review of clinical management protocols might have been more appropriate.

The themes and outcomes presented in the review relate more to leadership, coordination, and administrative aspects of healthcare. Aligning the title with the review question would provide a clearer and more accurate expectation for the reader. For example: Managing strategies to improve outcomes for pregnant women experiencing obstetric emergencies in sub-Saharan Africa.

Despite this misalignment, the strategies identified are valuable and significant for advancing midwifery and obstetric care for high-risk women.

Introduction

The introduction does not adequately highlight the existing gaps in the management of obstetric emergencies, nor does it clearly outline the strategies currently available to address these emergencies. As a result, the significance of this scoping review cannot be fully understood in relation to a clearly defined gap in the literature.

Methodology

The methodology is clearly described and aligns well with the structure of a typical scoping review. The PRISMA diagram and accompanying tables provide appropriate evidence of a systematic and comprehensive review process. However, the following shortcomings are noted:

Search Strategy

The search limiters and inclusion/exclusion criteria are not clearly described.

Results

The explanation of article exclusions and the rationale for these decisions is unclear, and the numerical flow should add up consistently. The authors should specify the exact reasons for excluding each group of articles.

In addition, the results should be presented in the same sequence as the themes outlined in the abstract, using consistent wording throughout. The themes are currently not discussed in a logical order, nor in the order in which they appear in the abstract. Furthermore, the discussion of these themes lacks sufficient detail:

4.2.1 Life-Saving Interventions

The narrative in section 4.2.1 should acknowledge that not all interventions resulted in improved maternal outcomes across all settings; several studies reported continued maternal mortality and complications despite the implementation of these interventions.

Table 4 includes not only clinical interventions but also system-level and triage-related strategies such as ambulance services [66], high-dependency unit (HDU) models [64], the MORES referral system [65], NMCR processes [67], the PPH emergency care package [52], and risk-stratification or monitoring tools including Shock Index thresholds [29], ICU utilisation profiles [38], pulmonary complication characterisation [37], near-miss incidence and EmOC signal functions [53], and ICU mortality prediction tools [35].

Suggestions to improve the results section:

- Explicitly group the evidence into clinical therapies, systems-strengthening interventions, and monitoring/triage tools to better reflect the breadth of content presented in Table 4. This will help readers understand that the theme encompasses more than clinical therapies—it also includes platform/system interventions and risk assessment tools.
- Consider adding sub-sections (or descriptors within the table) for:
  - Clinical therapies (NASG, $MgSO_4$, EPH),
  - Systems strengthening (ambulance services, HDU models, MORES, NMCR, PPH emergency care package),
  - Monitoring/triage tools (Shock Index, ICU utilisation/prediction metrics, pulmonary complication data, near-miss incidence and EmOC signal functions).

This structure should ideally be applied consistently across all themes and tables.

4.2.2 Training Interventions

- The narrative does not fully reflect the diversity of training interventions or the mixed outcomes presented in Table 5.
- Section 4.2.2 gives the impression of universally successful training outcomes, whereas Table 5 shows variable impact. For example, Germossa et al. [50] reported improved signal functions but unchanged mortality, and Okonofua et al. [33] found post-training knowledge scores below 46%, indicating persistent competency gaps.
- There is also a numerical inconsistency: the text reports n = 13 (consistent with Table 5), but only 11 studies are cited (26, 30, 32, 33, 39, 40, 50, 56, 61, 63, 68). Studies such as 69, 70, 71, and 72 are not referenced in the narrative.
- Additionally, the aims and outcomes of Nyamtema & Mtey (69) may not align well with this theme.

Suggestions:

- Introduce nuance in the narrative, for example: "While most training programmes improved providers' skills and confidence, several studies highlighted persistent knowledge gaps and limited impact on mortality outcomes."
- Expand the discussion to include continuing professional development (CPD) needs, digital and simulation-based innovations, and the variability in training effectiveness.

- Apply similar refinements to the narratives accompanying Tables 6 and 7 to ensure consistency, clarity, and accurate representation of the evidence.

Additional Recommendation

It would be beneficial to provide clear definitions of the key concepts used in the themes to enhance conceptual clarity and ensure consistent interpretation within the context of the scoping review.
