# Supplementary material for: Strategies to improve outcomes of obstetric emergencies in sub-Saharan Africa: A scoping review
Source: PLoS One. 2026 Sep 1;21(9):e0355174. doi: 10.1371/journal.pone.0355174 (PMC13533365; doi:10.1371/journal.pone.0355174)
Supplement: Supplementary file 2 [file pone.0355174.s005.pdf]

Strategies to improve the outcome of obstetric emergencies in sub-Saharan Africa: A scoping review

PONE-D-25-31834R1

Main claims of the paper and its significance for the discipline?

Obstetric emergencies—including postpartum haemorrhage, hypertensive disorders of pregnancy, sepsis, obstructed labour, and complications of unsafe abortion—remain a leading cause of maternal death in sub-Saharan Africa. Yet their effective management is undermined by incomplete EmONC functionality, workforce deficits, weak referral systems, and broader health-system failures, necessitating a scoping review to map existing strategies, expose persistent gaps, and guide contextually appropriate, multidimensional interventions.

The topic is relevant, and mapping the findings against existing strategies can significantly contribute to this speciality field and be used to improve the status quo.

A major weakness of the study is that the newly described strategies found in the literature have not been mapped to existing strategies. The scoping review, which also presented a mapping review, was an appropriate methodology for that.

Abstract

Line 38 – **The report** of the review followed the PRISMA guidelines.

The background can be reduced to provide a clearer overview of the methodology, e.g. data analysis. Technically, data synthesis for scoping reviews is incorrect, since data are rather descriptively mapped.

Aims and objectives

The objective is stated in the abstract as: This scoping review aimed to identify strategies reported in the literature to improve outcomes of obstetric emergencies in SSA. However, the aims and objectives are not described in the main manuscript.

The review question aligns with the objective stated in the abstract.

Introduction

Lines 63 and 79 & 79 seem repetitive. A similar comment applies to lines 64-65 and 80-82. Suggestion: paragraphs 1 and 2 can be combined into one concise paragraph.

Lines 78–81 need referencing.

Line 83 - Stats reference is outdated. There are much later WHO stats.

Line 85 needs a reference.

Line 87 is an incomplete sentence.

Lines 118-119 – describe the availability of these strategies and provide evidence on how they are implemented, adapted, and sustained.

119 across SSA remains fragmented.

Line 133 – the author should instead refer to the mapping of evidence. Evidence synthesis warrants a systematic review, which is a more specific methodology than a scoping review, which focuses specifically on mapping and describing evidence.

The introduction should conclude with an overarching review objective that captures and aligns with the core elements of your inclusion criteria, namely PCC. Revise lines 133-136.

Methodology

PCC well explained.

Note: the reviewer assessed the methodology against the theory of a scoping review, which differs from other types of reviews. There are some discrepancies with regard to the strategies used for data extraction and analysis. See the report under the following sections.

Search strategy

Lines 171-175 – the search terms are described in general, but the report should specify the actual key terms used to conduct the search. This is useful for replicating the search.

Study Selection

Report on full-text retrieval. Authors referred only to screening titles and abstracts. Details of the full-text articles retrieved should be provided.

There is no evidence of pilot testing of source selectors before embarking on source selection across the team.

Data extraction and quality assessment

Were the objectives, aims, or purpose of the studies examined?

Strategy for data synthesis

To preserve the characteristics of a pure scoping review and to distinguish it from systematic reviews or other reviews, use the “strategy to map evidence”. It is important to note that scoping reviews do not analyse through synthesis. Extracted data should be mapped and described against an “priori” set of data.

Results

Lines 224-225 and 265-266 are not included in the PRISMA diagram. Include.

Table 1 – include year of publication.

Line 276 – table name should be on top.

Table 3: Types of study designs reports only 54 studies, not 65. Complete the table.

Please state that the data was described in themes and not mapped against prior or existing strategies.

Contextual factors influencing outcomes of obstetric emergencies in SSA should be reported as additional or incidental outcomes, as they are not included in the objective stated in the abstract.

Recommendations

Clarify the aims and objectives in the main manuscript. Although the objective is stated in the abstract, it is absent from the body of the manuscript. A clearly articulated objective, aligned with the Population-Concept-Context (PCC) framework and the core inclusion criteria, should be included, and lines 133–136 should be revised accordingly.

Revise the introduction to eliminate repetition and update references. Lines 63, 79, and 80–82 contain redundant content and should be consolidated into a single concise paragraph. Outdated statistics (line 83) should be replaced with the most recent WHO figures. Missing references on lines 78–81, 85, and 118–119 should be added, and the incomplete sentence on line 87 should be corrected.

Reframe the language of the review throughout to reflect scoping review methodology. Terms such as "data synthesis" and "evidence synthesis" should be replaced with "mapping of evidence" or "descriptive mapping", as scoping reviews map and describe evidence rather than synthesise it. This applies to the abstract, methodology, and results sections.

Provide full details of the search strategy for replicability. Lines 171–175 describe the search terms only in general terms. The actual key terms, Boolean operators, and database-specific search strings used should be reported in full to enable independent replication of the search.

Report on full-text retrieval and pilot testing during study selection. The manuscript currently addresses only title and abstract screening. Details of the full-text articles retrieved and assessed for eligibility should be reported. Additionally, evidence of pilot testing the selection process among team members before full screening should be described.

Correct inconsistencies and omissions in the PRISMA diagram and tables. Lines 224–225 and 265–266 should be incorporated into the PRISMA diagram. Table 1 should include the year of publication, the table name in Table 3 (line 276) should be moved to the top, and Table 3 should be completed to account for all 65 studies rather than the 54 currently reported.

Clarify that the findings were described thematically rather than mapped against a priori strategies. The results section should explicitly state that the data were organised into themes rather than mapped against pre-existing or a priori strategies, thereby maintaining consistency with scoping review conventions and distinguishing the approach from systematic review methodology.

Reframe contextual factors as additional or incidental findings. Because contextual factors influencing outcomes of obstetric emergencies in SSA fall outside the objective stated in the abstract, they should be reported as supplementary or incidental findings rather than as primary results, ensuring alignment between the stated objective and the reported outcomes.
