# Supplementary material for: Strategies to improve outcomes of obstetric emergencies in sub-Saharan Africa: A scoping review
Source: PLoS One. 2026 Sep 1;21(9):e0355174. doi: 10.1371/journal.pone.0355174 (PMC13533365; doi:10.1371/journal.pone.0355174)
Supplement: Supplementary file 3 [file pone.0355174.s001.docx]

**S1 File. Search strategies used for the electronic databases.**

| **Database** | **Search query** | **Limits** | **Results** |
| --- | --- | --- | --- |
| PubMed | (("strategie"[All Fields] OR "strategies"[All Fields] OR "strategy"[All Fields] OR "strategy s"[All Fields] OR ("strategie"[All Fields] OR "strategies"[All Fields] OR "strategy"[All Fields] OR "strategy s"[All Fields]) OR "Plan"[All Fields] OR ("policy"[MeSH Terms] OR "policy"[All Fields] OR "policies"[All Fields] OR "policy s"[All Fields]) OR ("approach"[All Fields] OR "approach s"[All Fields] OR "approachability"[All Fields] OR "approachable"[All Fields] OR "approache"[All Fields] OR "approached"[All Fields] OR "approaches"[All Fields] OR "approaching"[All Fields] OR "approachs"[All Fields]) OR ("methods"[MeSH Terms] OR "methods"[All Fields] OR "procedure"[All Fields] OR "methods"[MeSH Subheading] OR "procedures"[All Fields] OR "procedural"[All Fields] OR "procedurally"[All Fields] OR "procedure s"[All Fields])) AND ((("obstetric"[All Fields] OR "obstetrically"[All Fields] OR "obstetrics"[MeSH Terms] OR "obstetrics"[All Fields] OR "obstetrical"[All Fields] OR ("perinatal"[All Fields] OR "perinatally"[All Fields] OR "perinatals"[All Fields])) AND "Critical Care"[All Fields]) OR "Emergency Care"[All Fields] OR "Acute Care"[All Fields] OR "Intensive Care"[All Fields] OR "Obstetric care"[All Fields] OR "perinatal care"[All Fields]) AND ("Maternal Health"[All Fields] OR "Maternal Mortality"[All Fields] OR "Maternal Death"[All Fields]) AND "Africa South of the Sahara"[MeSH Terms]) AND ((english[Filter]) AND (2019:2025[pdat])) | *Year 2019-2025*  *Language; English* | 270 |
| CINAHL | TX ( Strategy OR Strategies OR Plan OR Policy or Approach OR Procedure ) AND TX ( Obstetric OR Perinatal AND “Critical Care” OR “Emergency Care” OR “Acute Care” OR “Intensive Care” OR “Obstetric care” OR “perinatal care” ) AND TX ( “Maternal Health” OR “Maternal Mortality” OR “Maternal Death” ) | - Published Date: 20190101-20251231 - Geography - africa | 230 |
| Scopus | ( TITLE-ABS-KEY ( critical AND care ) AND TITLE-ABS-KEY ( obstetric AND emergencies ) AND AFFILCOUNTRY ( "Angola" OR "Benin" OR "Botswana" OR "Burkina Faso" OR "Burundi" OR "Cameroon" OR "Chad" OR "Congo" OR "Cote d'Ivoire" OR "Democratic Republic Congo" OR "Ethiopia" OR "Gabon" OR "Gambia" OR "Ghana" OR "Guinea" OR "Guinea-Bissau" OR "Kenya" OR "Liberia" OR "Madagascar" OR "Malawi" OR "Mali" OR "Mauritania" OR "Mauritius" OR "Mozambique" OR "Namibia" OR "Niger" OR "Nigeria" OR "Rwanda" OR "Senegal" OR "Sierra Leone" OR "South Africa" OR "Sudan" OR "Swaziland" OR "Tanzania" OR "Uganda" OR "Zambia" OR "Zimbabwe" ) ) AND PUBYEAR > 2018 AND PUBYEAR < 2026 | *Year 2019-2025*  *Language; English* | 79 |
| CoRe | Strategy OR Strategies OR Plan OR Policy or Approach OR Procedure AND Obstetric OR Perinatal AND “Critical Care” OR “Emergency Care” OR “Acute Care” OR “Intensive Care” OR “Obstetric care” OR “perinatal care” AND “Maternal Health” OR “Maternal Mortality” OR “Maternal Death” AND Africa | *Year 2019-2025* | 149 |
| TOTAL | The database searches retrieved 728 records. After export into EndNote and removal of duplicate records, 472 unique records were retained for title and abstract screening. |  | 728 |
