# Supplementary material for: Strategies to improve outcomes of obstetric emergencies in sub-Saharan Africa: A scoping review
Source: PLoS One. 2026 Sep 1;21(9):e0355174. doi: 10.1371/journal.pone.0355174 (PMC13533365; doi:10.1371/journal.pone.0355174)
Supplement: Supplementary file 4 [file pone.0355174.s002.docx]

PRISMA Extension for Scoping Reviews (PRISMA-ScR) S1_Checklist

**Manuscript: Strategies to improve outcomes of obstetric emergencies in sub-Saharan Africa**

| **Section** | **Item** | **Reporting item** | **Location in manuscript** |
| --- | --- | --- | --- |
| **TITLE** | 1 | Identify the report as a scoping review. | Title page, p. 1 |
| **ABSTRACT** | 2 | Provide a structured summary covering the background, objectives, eligibility criteria, information sources, charting methods, results, and conclusions. | Abstract, p. 2 |
| **INTRODUCTION** | 3 | Explain the rationale for the review in relation to existing knowledge and why a scoping review approach is appropriate. | Introduction, pp. 3–6 |
| **INTRODUCTION** | 4 | State the review objectives and questions, including the population, concept, and context. | Introduction and Review question, p. 6 |
| **METHODS** | 5 | State whether a protocol exists and provide registration or access information when applicable. | Not reported in the manuscript |
| **METHODS** | 6 | Specify the eligibility criteria and explain how they were applied. | Eligibility criteria, pp. 6–7 |
| **METHODS** | 7 | Describe all information sources and the date range covered by the search. | Search Strategy, p. 7; S1 File |
| **METHODS** | 8 | Present a reproducible electronic search strategy for at least one database, including limits used. | Search Strategy, p. 7; complete strategies in S1 File |
| **METHODS** | 9 | Describe the process used to select sources of evidence at each screening stage. | Study Selection, p. 7 |
| **METHODS** | 10 | Describe the data-charting process, including the charting form, reviewers involved, and how disagreements were handled. | Data extraction and quality assessment, pp. 7–8 |
| **METHODS** | 11 | List and define the variables and other information extracted from included sources. | Data extraction and quality assessment, pp. 7–8 |
| **METHODS** | 12 | If critical appraisal was performed, explain why and describe the methods used. | Not applicable; formal critical appraisal was not undertaken. Rationale provided under Quality assessment, p. 8 |
| **METHODS** | 13 | Describe the methods used to organise, map, and summarise the charted data. | Strategy for mapping the evidence, p. 8 |
| **RESULTS** | 14 | Report the number of records screened, assessed, and included, with reasons for full-text exclusions; present a flow diagram where appropriate. | Results and Fig 1 caption, pp. 8–9; Fig1.tif |
| **RESULTS** | 15 | Present the characteristics of included sources and cite them. | Characteristics of included studies and Tables 1–3, pp. 9–45 |
| **RESULTS** | 16 | If critical appraisal was performed, report the appraisal results. | Not applicable; critical appraisal was not undertaken |
| **RESULTS** | 17 | Present the relevant charted data for each included source. | Table 1, pp. 10–44 |
| **RESULTS** | 18 | Summarise and map the results in relation to the review objectives and questions. | Thematic analysis and Tables 4–5, pp. 46–63 |
| **DISCUSSION** | 19 | Summarise the principal findings, themes, evidence types, and gaps, and discuss their relevance. | Discussion, pp. 64–68 |
| **DISCUSSION** | 20 | Discuss limitations of the scoping review process. | Strengths and Limitations, p. 68 |
| **DISCUSSION** | 21 | Provide an overall interpretation and discuss implications or recommended next steps. | Conclusion, implications, and recommendations, pp. 68–71 |
| **FUNDING** | 22 | Describe funding for the scoping review and, when charted, funding of included sources; state the role of funders. | Review funding declaration provided in the online submission system; funding of included sources was not charted |

**Reference:** Tricco AC, et al. PRISMA Extension for Scoping Reviews (PRISMA-ScR): Checklist and Explanation. Ann Intern Med. 2018;169:467–473. doi:10.7326/M18-0850.
