# Supplementary material for: Strategies to improve outcomes of obstetric emergencies in sub-Saharan Africa: A scoping review
Source: PLoS One. 2026 Sep 1;21(9):e0355174. doi: 10.1371/journal.pone.0355174 (PMC13533365; doi:10.1371/journal.pone.0355174)
Supplement: Supplementary file 5 [file pone.0355174.s004.docx]

# Response to Reviewers

Manuscript Title: Strategies to improve outcomes of obstetric emergencies in sub-Saharan Africa: A scoping review

Dear Editor and Reviewers,

We sincerely thank the Editor and Reviewers for their thoughtful and constructive feedback. All comments have been carefully addressed. Revisions are visible in the revised manuscript using tracked changes. Below is a detailed point‑by‑point response following PLOS ONE formatting guidance.

| **Reviewer Comment** | **Author Response** | **Location in Revised Manuscript** |
| --- | --- | --- |
| Title does not align with findings. | The title has been revised to reflect the integrated and multi‑level strategies identified (clinical, workforce, health‑system, referral, and contextual). This change is visible in tracked changes and aligns the title with thematic synthesis. | Title page; Abstract; Introduction |
| Justification for article exclusions unclear; numerical flow inconsistent. | The PRISMA description and Results text were rewritten to provide explicit screening numbers and precise exclusion reasons. All numerical inconsistencies were corrected in tracked changes. | Methods – Study selection; Results; Figure 1 |
| Table 1 not referenced in text. | A narrative reference to Table 1 was inserted in the Results section describing study characteristics. This insertion is shown in tracked changes. | Results – Characteristics of included studies |
| Maternal mortality data outdated. | All epidemiological statistics were updated to the most recent WHO and global estimates. Replacements are visible in tracked changes. | Introduction; Discussion |
| Improve logical flow of Introduction and clarify knowledge gap. | The Introduction was reorganised to follow a structured sequence: global burden → SSA disparity → existing strategies → knowledge gap → rationale for the review. A new paragraph explicitly describing the literature gap was added in tracked changes. | Introduction |
| Search strategy timing and update unclear. | Methods now specify databases, eligibility criteria, original date range (2019–2024), and targeted update to January 2026 incorporating additional eligible studies. These edits appear in tracked changes. | Methods – Search strategy; Results |
| Results should follow thematic order of abstract. | Results and Discussion were reorganised to consistent thematic sequence: (1) life‑saving interventions, (2) workforce training, (3) health‑system/referral/policy, (4) contextual barriers. Structural edits are visible in tracked changes. | Abstract; Results; Discussion |
| Life‑saving interventions overstated. | Section 4.2.1 was revised to explicitly acknowledge heterogeneous effectiveness and persistent maternal mortality in some settings. Revised interpretation appears in tracked changes. | Results – Theme 1 |
| Training interventions presented as uniformly successful. | Section 4.2.2 was rewritten to include all relevant studies, correct citation inconsistencies, and explicitly report mixed outcomes and persistent competency gaps. Changes are visible in tracked changes. | Results – Theme 2 |
| Strengths and limitations are insufficient | The Strengths and Limitations section was expanded to discuss geographic, temporal, and language restrictions and inherent scoping‑review methodological limits. Added text is shown in tracked changes. | Discussion – Strengths & Limitations |
| Separate conclusions from implications. | The final section was divided into two subsections: Conclusions and Implications for policy, practice, and research. Structural edits are visible in tracked changes. | Conclusion; Implications section |
| Data availability concerns. | The Data Availability Statement was revised to comply fully with PLOS ONE open‑data policy. Updated wording appears in tracked changes. | Data Availability Statement |

We are grateful for the reviewers’ constructive guidance, which substantially strengthened the clarity, methodological rigor, and policy relevance of this manuscript. We respectfully submit the revised manuscript for reconsideration in PLOS ONE.

Kind regards,
Dr. Serah Wachira
(On behalf of all authors)
