# Supplementary material for: Strategies to improve outcomes of obstetric emergencies in sub-Saharan Africa: A scoping review
Source: PLoS One. 2026 Sep 1;21(9):e0355174. doi: 10.1371/journal.pone.0355174 (PMC13533365; doi:10.1371/journal.pone.0355174)
Supplement: Supplementary file 6 [file pone.0355174.s006.docx]

**Response to Reviewers**

**Manuscript Title:** *Strategies to Improve Outcomes of Obstetric Emergencies in Sub-Saharan Africa: A Scoping Review*

**Manuscript ID:** PONE-D-25-31834R1

We sincerely thank the Editor and Reviewer for the insightful and constructive comments. We have carefully considered each recommendation and revised the manuscript accordingly. All changes have been incorporated into the revised manuscript and highlighted for ease of review.

| **Reviewer Comment** | **Response** | **Location in Revised Manuscript** |
| --- | --- | --- |
| Major comment: The newly described strategies have not been mapped to existing strategies. | Thank you for this important observation. The manuscript has been substantially revised to explicitly map the identified strategies into three overarching strategy domains: (1) life-saving clinical and health-system interventions, (2) health workforce education, training and mentorship, and (3) health system organisation, referral mechanisms and policy implementation. A summary mapping table (Table 4) has been added to provide an overview of the strategy domains. | Results (Thematic Analysis), Table 4 |
| Replace "data synthesis" with terminology appropriate for a scoping review. | Revised throughout the manuscript. Terminology has been changed from "data synthesis" and "evidence synthesis" to "mapping of evidence," "descriptive thematic analysis," and "strategy for mapping the evidence," consistent with PRISMA-ScR methodology. | Abstract, Methods, Results |
| The abstract background is too long. | The abstract has been substantially condensed to improve balance between background, methods, results, and conclusions. | Abstract |
| The abstract objective should align with the manuscript objective. | The objective has been revised for consistency and is now presented identically in both the abstract and the main manuscript. | Abstract; Introduction |
| The aims and objectives are missing from the main manuscript. | A clearly articulated review objective aligned with the Population–Concept–Context (PCC) framework has been added at the end of the Introduction. | Introduction |
| Introduction contains repetitive paragraphs. | Repetitive content has been consolidated and the Introduction rewritten to improve flow and reduce redundancy. | Introduction |
| Update outdated WHO statistics and references. | Maternal mortality statistics have been updated using the most recent WHO publications, and outdated references have been replaced. | Introduction |
| Add missing references. | Missing references have been added throughout the Introduction and Background where appropriate. | Introduction |
| Incomplete sentence. | Corrected. | Introduction |
| Describe how strategies are implemented, adapted and sustained. | Additional discussion has been incorporated into the Background and Discussion describing implementation, adaptation and sustainability of obstetric emergency strategies. | Introduction; Discussion |
| Search strategy should report actual search terms. | The search strategy has been expanded to describe the controlled vocabulary (MeSH) and free-text keywords used. Complete database-specific search strings are provided in Supplementary File 1. | Methods—Search Strategy; Supplementary File 1 |
| Report full-text retrieval. | The study selection section has been revised to describe title/abstract screening, full-text retrieval, full-text assessment, and reasons for exclusion. These are also reflected in the PRISMA flow diagram. | Methods; Figure 1 |
| No evidence of pilot testing during screening. | A description has been added explaining that all reviewers participated in an online calibration meeting before screening to review the screening tool, discuss PCC eligibility criteria, and ensure consistent application of the inclusion and exclusion criteria. | Methods—Study Selection |
| Were study objectives extracted? | Yes. The data extraction section has been revised to specify that study objective/aim was extracted together with study characteristics, interventions, implementation context, and reported outcomes. | Methods—Data Extraction |
| Use "Strategy for mapping evidence" instead of "Strategy for data synthesis." | The section has been renamed Strategy for Mapping the Evidence, and the description revised to reflect descriptive thematic mapping consistent with scoping review methodology. | Methods |
| PRISMA diagram incomplete. | The PRISMA flow diagram has been updated to include the complete screening process, full-text exclusions with reasons, and final study numbers. | Figure 1 |
| Table 1 should include publication year. | Publication year has been added for all included studies. | Table 1 |
| Table title placement. | Corrected. | Table 3 |
| Table 3 study numbers incomplete. | Table 3 has been reconstructed and now includes all included studies (n = 64). | Table 3 |
| State that findings were described thematically rather than mapped against existing strategies. | The Results have been revised to clarify that findings were organised using descriptive thematic analysis. Three themes describe the mapped strategies, while contextual factors are presented as additional findings influencing implementation. | Results |
| Contextual factors should be presented as additional findings rather than primary themes. | We agree. The manuscript has been revised accordingly. Contextual factors are now presented under Additional Findings: Contextual Factors Influencing the Implementation of Strategies, consistent with the review objective. Table 7 and the corresponding narrative have been revised to reflect this change. | Results; Discussion; Table 8 |
| Clarify consistency between objectives, results and discussion. | The objective, thematic organisation, Discussion, Implications, Recommendations, and Conclusion have all been revised to ensure complete alignment throughout the manuscript. | Throughout manuscript |

We sincerely thank the reviewer for the thoughtful and constructive feedback. The comments have substantially strengthened the manuscript by improving its methodological alignment with PRISMA-ScR guidance, clarifying the thematic organization, strengthening the interpretation of findings, and ensuring consistency across the abstract, methods, results, discussion, and conclusion. We believe the revised manuscript addresses all reviewer concerns and is considerably improved as a result.
