# Supplementary material for: Strategies to improve outcomes of obstetric emergencies in sub-Saharan Africa: A scoping review
Source: PLoS One. 2026 Sep 1;21(9):e0355174. doi: 10.1371/journal.pone.0355174 (PMC13533365; doi:10.1371/journal.pone.0355174)
Supplement: Peer review history

# Peer review: Decision Letter 0

**DOI:** 10.1371/journal.pone.0355174.r001

**Authors:** Maurine Rofhiwa Musie

10 Dec 2025

Dear Dr. Wachira,

Thank you for submitting your manuscript to PLOS ONE. After careful consideration, we feel that it has merit but does not fully meet PLOS ONE’s publication criteria as it currently stands. Therefore, we invite you to submit a revised version of the manuscript that addresses the points raised during the review process.

==============================

**ACADEMIC EDITOR:**

After careful evaluation of the reviewers’ reports, we have concluded that your manuscript may be suitable for publication **pending major revision** . Both reviewers acknowledge the relevance and potential contribution of your work; however, they have recommended several important revisions to strengthen the methodological rigour, clarity of argumentation, and overall coherence of the manuscript.

- *The title of the scoping review does not align with the findings presented in the results section.*
- *The methodology section requires restructuring for clarity and coherence.*
- *The justification for article exclusions is insufficiently explained, and the numerical flow lacks consistency. The authors should clearly outline the specific reasons for excluding each category of articles, ensuring that all numbers correspond accurately throughout the review*

Please submit your revised manuscript by Jan 24 2026 11:59PM. If you will need more time than this to complete your revisions, please reply to this message or contact the journal office at plosone@plos.org. When you're ready to submit your revision, log on to https://www.editorialmanager.com/pone/ and select the 'Submissions Needing Revision' folder to locate your manuscript file.

- A rebuttal letter that responds to each point raised by the academic editor and reviewer(s). You should upload this letter as a separate file labeled 'Response to Reviewers'.
- A marked-up copy of your manuscript that highlights changes made to the original version. You should upload this as a separate file labeled 'Revised Manuscript with Track Changes'.
- An unmarked version of your revised paper without tracked changes. You should upload this as a separate file labeled 'Manuscript'.

If you would like to make changes to your financial disclosure, please include your updated statement in your cover letter. Guidelines for resubmitting your figure files are available below the reviewer comments at the end of this letter.

If applicable, we recommend that you deposit your laboratory protocols in protocols.io to enhance the reproducibility of your results. Protocols.io assigns your protocol its own identifier (DOI) so that it can be cited independently in the future. For instructions see: https://journals.plos.org/plosone/s/submission-guidelines#loc-laboratory-protocols. Additionally, PLOS ONE offers an option for publishing peer-reviewed Lab Protocol articles, which describe protocols hosted on protocols.io. Read more information on sharing protocols at https://plos.org/protocols?utm_medium=editorial-email&utm_source=authorletters&utm_campaign=protocols.

We look forward to receiving your revised manuscript.

Kind regards,

Maurine Rofhiwa Musie, PhD

Academic Editor

PLOS One

Journal Requirements:

When submitting your revision, we need you to address these additional requirements.

1. Please ensure that your manuscript meets PLOS ONE's style requirements, including those for file naming. The PLOS ONE style templates can be found at

https://journals.plos.org/plosone/s/file?id=wjVg/PLOSOne_formatting_sample_main_body.pdf and

https://journals.plos.org/plosone/s/file?id=ba62/PLOSOne_formatting_sample_title_authors_affiliations.pdf

2. When completing the data availability statement of the submission form, you indicated that you will make your data available on acceptance. We strongly recommend all authors decide on a data sharing plan before acceptance, as the process can be lengthy and hold up publication timelines. Please note that, though access restrictions are acceptable now, your entire data will need to be made freely accessible if your manuscript is accepted for publication. This policy applies to all data except where public deposition would breach compliance with the protocol approved by your research ethics board. If you are unable to adhere to our open data policy, please kindly revise your statement to explain your reasoning and we will seek the editor's input on an exemption. Please be assured that, once you have provided your new statement, the assessment of your exemption will not hold up the peer review process.

3. We note you have included a table to which you do not refer in the text of your manuscript. Please ensure that you refer to Table 1 in your text; if accepted, production will need this reference to link the reader to the Table.

4. If the reviewer comments include a recommendation to cite specific previously published works, please review and evaluate these publications to determine whether they are relevant and should be cited. There is no requirement to cite these works unless the editor has indicated otherwise.

[Note: HTML markup is below. Please do not edit.]

Reviewers' comments:

Reviewer's Responses to Questions

**Comments to the Author**

1. Is the manuscript technically sound, and do the data support the conclusions?

Reviewer #1: Yes

Reviewer #2: Partly

**********

2. Has the statistical analysis been performed appropriately and rigorously?

Reviewer #1: N/A

Reviewer #2: Yes

**********

3. Have the authors made all data underlying the findings in their manuscript fully available?

The PLOS Data policy

Reviewer #1: Yes

Reviewer #2: No

**********

4. Is the manuscript presented in an intelligible fashion and written in standard English?

Reviewer #1: Yes

Reviewer #2: Yes

**********

Reviewer #1: Dear Authors,

It was a pleasure reviewing your work. I appreciate the efforts put into this manuscript that addresses an important area of work in maternal health.

I have raised a few queries and made some suggestions which I believe could significantly improve the quality, rigour, and impact of the paper.

I would be happy to review your revised manuscript and responses.

Kind regards,

Reviewer

Detailed feedback.

Scope of the Search

Individual country names:

Including individual country names in your search strategy instead of just “Africa South of the Sahara” could have improved your search.

Use of the term “Sub-Saharan Africa”:

The term “Sub-Saharan Africa” has been critiqued for its colonial and exclusionary connotations. You may wish to consider using "Africa" instead, or be explicit about which countries or regions are included and why. See: https://readcultured.com/its-time-to-drop-sub-saharan-africa-from-our-vocabulary-3cc0561a57cb

Additionally, maternal mortality remains a challenge in several African countries not commonly categorized as sub-Saharan e.g. Sudan

Timeframe of included studies (2019–2024):

The rationale for limiting the search to this recent window is unclear. For a scoping review to be useful for informing policy, research, and practice, it may benefit from a broader historical scope, including key milestones such as the Safe Motherhood Initiative (1987), the Millennium Development Goals, and the Sustainable Development Goals. Recency is valuable, but comprehensiveness is critical.

Language limitations:

Restricting the search to English-language studies may bias the review. Many African countries where the problem of maternal mortality is high, publish in French or Portuguese and excluding these may miss relevant data.

Introduction

“Low- and middle-income countries”:

This is a World Bank income classification that changes over time and should be used carefully. Consider also aligning with prior comments about terminology and geographical scope.

Evidence base:

Please provide a citation to support the statement: “Despite global efforts, the burden of maternal mortality continues to be disproportionately high in these regions.” I suggest referencing:

The Lancet Global Health 2024 article

https://www.thelancet.com/journals/langlo/article/PIIS2214-109X(24)00560-6/fulltext

WHO Trends in Maternal Mortality 2024

https://iris.who.int/handle/10665/381012

Outdated data:

The citation of maternal mortality data from 2017 (WHO) is outdated. Please refer to more recent estimates as per the above resources.

Please check all the references – to ensure that they are the most recent and the most relevant to your work. I only highlighted 2.

Structure and flow:

Consider reviewing the paragraph structures to enhance logical flow—ideally moving from the problem statement, to significance, existing gaps, and finally the rationale for this review.

Methods

These are largely well described and can be replicated.

Typographical error:

It is “Joanna Briggs Institute,” not “Joan Brigg’s”—please correct.

Search strategy:

When was the literature search conducted? Was it updated just incase new articles were published after the initial search? – if so, please indicate.

Results

Characteristics of included studies:

The sentence “Tables 2 and 3 summarize the key attributes…” is descriptive but does not reflect synthesis.

Consider beginning the section with a synthesis of study characteristics—highlighting common designs, settings, and participant types—before referencing the tables.

Some of the narrative synthesis appears after the tables. Reorganizing this so that a narrative introduction precedes the tables would improve clarity and flow. Table 1 should follow the narrative synthesis rather than precede it. (This links to my comment above)

Consider reducing emphasis on publication year unless relevant (e.g., if it was bibliometric analysis). Instead, focus on study characteristics like study location, population, interventions, outcomes, and settings – A synthesis not a replication of the content in table 1.

Thematic synthesis:

This section is well structured and clearly written. It reflects a good understanding of the findings.

Strengths and Limitations

This section could be strengthened by including a more critical appraisal of the limitations of your search strategy, particularly the geographic, temporal, and language scope that I highlighted earlier. Especially if you choose to leave the scoping review as it is.

Conclusions and Recommendations

Consider separating this into two distinct sections:

Conclusions (to summarise the findings)

Implications for policy, research, and practice (to enhance clarity and structure)

In summary,

This is an important and timely topic, and with a few adjustments in scope, structure, and terminology, the paper could make a valuable contribution to the field.

Reviewer #2: 1) The Title must be aligned with the content of the article.

2) Concepts in the themes must be defined for clarity.

3) The narratives of the tables (discussion of results) need a detailed description.

4) The introduction should highlight existing strategies and point out the gap that steered this scoping review.

**********

what does this mean?). If published, this will include your full peer review and any attached files.

If you choose “no”, your identity will remain anonymous but your review may still be made public.

**Do you want your identity to be public for this peer review?**  For information about this choice, including consent withdrawal, please see our Privacy Policy

Reviewer #1: **Yes:** Francis G Muriithi, MBChB, MMed O&G, MSc EBHC, MRCOG (UK).

Reviewer #2: No

**********

[NOTE: If reviewer comments were submitted as an attachment file, they will be attached to this email and accessible via the submission site. Please log into your account, locate the manuscript record, and check for the action link "View Attachments". If this link does not appear, there are no attachment files.]

To ensure your figures meet our technical requirements, please review our figure guidelines: https://journals.plos.org/plosone/s/figures

You may also use PLOS’s free figure tool, NAAS, to help you prepare publication quality figures: https://journals.plos.org/plosone/s/figures#loc-tools-for-figure-preparation.

NAAS will assess whether your figures meet our technical requirements by comparing each figure against our figure specifications.

Attachment Submitted filename: REVIEWER REPORT.pdf

# Author comment: Author response to Decision Letter 1

**DOI:** 10.1371/journal.pone.0355174.r002

7 Feb 2026

We thank the editors and reviewers for their constructive and detailed feedback. All reviewer and editor comments have been carefully addressed, and the manuscript has been revised accordingly.

A detailed, point-by-point response to each comment is provided in the uploaded response-to-reviewers document. All revisions have been incorporated into the revised manuscript, and changes have been clearly indicated.

Attachment Submitted filename: Response to Reviewers PLOS One Manuscript 6.2.26.docx

# Peer review: Decision Letter 1

**DOI:** 10.1371/journal.pone.0355174.r003

**Authors:** Sabita Tuladhar

3 Jun 2026

Dear Dr. Wachira,

Thank you for addressing the comments. There are a few additional suggestions that could further strengthen the paper.

Please submit your revised manuscript by Jul 18 2026 11:59PM. If you will need more time than this to complete your revisions, please reply to this message or contact the journal office at plosone@plos.org. When you're ready to submit your revision, log on to https://www.editorialmanager.com/pone/ and select the 'Submissions Needing Revision' folder to locate your manuscript file.

- A letter that responds to each point raised by the academic editor and reviewer(s). You should upload this letter as a separate file labeled 'Response to Reviewers'.
- A marked-up copy of your manuscript that highlights changes made to the original version. You should upload this as a separate file labeled 'Revised Manuscript with Track Changes'.
- An unmarked version of your revised paper without tracked changes. You should upload this as a separate file labeled 'Manuscript'.

If you would like to make changes to your financial disclosure, please include your updated statement in your cover letter. Guidelines for resubmitting your figure files are available below the reviewer comments at the end of this letter.

If applicable, we recommend that you deposit your laboratory protocols in protocols.io to enhance the reproducibility of your results. Protocols.io assigns your protocol its own identifier (DOI) so that it can be cited independently in the future. For instructions see: https://journals.plos.org/plosone/s/submission-guidelines#loc-laboratory-protocols. Additionally, PLOS ONE offers an option for publishing peer-reviewed Lab Protocol articles, which describe protocols hosted on protocols.io. Read more information on sharing protocols at https://plos.org/protocols?utm_medium=editorial-email&utm_source=authorletters&utm_campaign=protocols.

As the corresponding author, your ORCID iD is verified in the submission system and will appear in the published article. PLOS supports the use of ORCID, and we encourage all coauthors to register for an ORCID iD and use it as well. Please encourage your coauthors to verify their ORCID iD within the submission system before final acceptance, as unverified ORCID iDs will not appear in the published article. *Only*  the individual author can complete the verification step; PLOS staff *cannot*  verify ORCID iDs on behalf of authors.

We look forward to receiving your revised manuscript.

Kind regards,

Sabita Tuladhar, PhD, MHealSc, MA

Academic Editor

PLOS One

Journal Requirements:

1. If the reviewer comments include a recommendation to cite specific previously published works, please review and evaluate these publications to determine whether they are relevant and should be cited. There is no requirement to cite these works unless the editor has indicated otherwise.

2. Please review your reference list to ensure that it is complete and correct. If you have cited papers that have been retracted, please include the rationale for doing so in the manuscript text, or remove these references and replace them with relevant current references. Any changes to the reference list should be mentioned in the rebuttal letter that accompanies your revised manuscript. If you need to cite a retracted article, indicate the article’s retracted status in the References list and also include a citation and full reference for the retraction notice.

[Note: HTML markup is below. Please do not edit.]

Reviewers' comments:

Reviewer's Responses to Questions

**Comments to the Author**

Reviewer #2: (No Response)

**********

2. Is the manuscript technically sound, and do the data support the conclusions?

Reviewer #2: Yes

**********

3. Has the statistical analysis been performed appropriately and rigorously?

Reviewer #2: Yes

**********

4. Have the authors made all data underlying the findings in their manuscript fully available?

The PLOS Data policy

Reviewer #2: Yes

**********

5. Is the manuscript presented in an intelligible fashion and written in standard English?

Reviewer #2: Yes

**********

Reviewer #2: Major corrections were addressed, but the reviewer still finds additional issues to correct before the manuscript is sound for publication.

**********

what does this mean?). If published, this will include your full peer review and any attached files.

If you choose “no”, your identity will remain anonymous but your review may still be made public.

**Do you want your identity to be public for this peer review?**  For information about this choice, including consent withdrawal, please see our Privacy Policy

Reviewer #2: No

**********

[NOTE: If reviewer comments were submitted as an attachment file, they will be attached to this email and accessible via the submission site. Please log into your account, locate the manuscript record, and check for the action link "View Attachments". If this link does not appear, there are no attachment files.]

To ensure your figures meet our technical requirements, please review our figure guidelines: https://journals.plos.org/plosone/s/figures

You may also use PLOS’s free figure tool, NAAS, to help you prepare publication quality figures: https://journals.plos.org/plosone/s/figures#loc-tools-for-figure-preparation.

NAAS will assess whether your figures meet our technical requirements by comparing each figure against our figure specifications.

Attachment Submitted filename: REVIEWER REPORT PONE-D-25-31834R1.pdf

# Author comment: Author response to Decision Letter 2

**DOI:** 10.1371/journal.pone.0355174.r004

14 Jul 2026

We sincerely thank the reviewer for the thoughtful and constructive feedback. The comments have substantially strengthened the manuscript by improving its methodological alignment with PRISMA-ScR guidance, clarifying the thematic organization, strengthening the interpretation of the findings, and ensuring consistency across the abstract, methods, results, discussion, and conclusion. We believe the revised manuscript addresses all reviewer comments and is considerably improved as a result. A detailed, point-by-point response to each reviewer comment is provided in the attached Response to Reviewers document.

Attachment Submitted filename: Response to Reviewers 7.7.26.docx

# Editor report: Decision Letter 2

**DOI:** 10.1371/journal.pone.0355174.r005

**Authors:** Sabita Tuladhar

20 Jul 2026

Strategies to improve the outcome of obstetric emergencies in sub-Saharan Africa: A scoping review

PONE-D-25-31834R2

Dear Dr. Wachira,

We’re pleased to inform you that your manuscript has been judged scientifically suitable for publication and will be formally accepted for publication once it meets all outstanding technical requirements.

Within one week, you’ll receive an e-mail detailing the required amendments. When these have been addressed, you’ll receive a formal acceptance letter and your manuscript will be scheduled for publication.

An invoice will be generated when your article is formally accepted. Please note, if your institution has a publishing partnership with PLOS and your article meets the relevant criteria, all or part of your publication costs will be covered. Please make sure your user information is up-to-date by logging into Editorial Manager at Editorial Manager® and clicking the ‘Update My Information' link at the top of the page. For questions related to billing, please contact billing support.

If your institution or institutions have a press office, please notify them about your upcoming paper to help maximize its impact. If they’ll be preparing press materials, please inform our press team as soon as possible -- no later than 48 hours after receiving the formal acceptance. Your manuscript will remain under strict press embargo until 2 pm Eastern Time on the date of publication. For more information, please contact onepress@plos.org.

Kind regards,

Sabita Tuladhar, PhD, MHealSc, MA

Academic Editor

PLOS One

Additional Editor Comments (optional): The authors have responded satisfactorily to all comments.

Reviewers' comments: None.

# Editor report: Acceptance letter

**DOI:** 10.1371/journal.pone.0355174.r006

**Authors:** Sabita Tuladhar

PONE-D-25-31834R2

PLOS One

Dear Dr. Wachira,

I'm pleased to inform you that your manuscript has been deemed suitable for publication in PLOS One. Congratulations! Your manuscript is now being handed over to our production team.

At this stage, our production department will prepare your paper for publication. This includes ensuring the following:

* All references, tables, and figures are properly cited

* All relevant supporting information is included in the manuscript submission,

* There are no issues that prevent the paper from being properly typeset

You will receive further instructions from the production team, including instructions on how to review your proof when it is ready. Please keep in mind that we are working through a large volume of accepted articles, so please give us a few days to review your paper and let you know the next and final steps.

Lastly, if your institution or institutions have a press office, please let them know about your upcoming paper now to help maximize its impact. If they'll be preparing press materials, please inform our press team within the next 48 hours. Your manuscript will remain under strict press embargo until 2 pm Eastern Time on the date of publication. For more information, please contact onepress@plos.org.

You will receive an invoice from PLOS for your publication fee after your manuscript has reached the completed accept phase. If you receive an email requesting payment before acceptance or for any other service, this may be a phishing scheme. Learn how to identify phishing emails and protect your accounts at https://explore.plos.org/phishing.

If we can help with anything else, please email us at customercare@plos.org.

Thank you for submitting your work to PLOS One and supporting open access.

Kind regards,

PLOS One Editorial Office Staff

on behalf of

Dr. Sabita Tuladhar

Academic Editor

PLOS One
